# Pediatric Blood Pressure and Cardiovascular Health in Adulthood

**DOI:** 10.1007/s11906-024-01312-5

**Published:** 2024-06-15

**Authors:** Yaxing Meng, Jonathan P. Mynard, Kylie J. Smith, Markus Juonala, Elaine M. Urbina, Teemu Niiranen, Stephen R. Daniels, Bo Xi, Costan G. Magnussen

**Affiliations:** 1 Baker Heart and Diabetes Institute, 75 Commercial Rd, Melbourne, VIC 3004 Australia; 2 Baker Department of Cardiometabolic Health, Faculty of Medicine, Dentistry and Health Sciences, University of Melbourne, Melbourne, VIC Australia; 3 Heart Research Group, Murdoch Childrenʼs Research Institute, Parkville, VIC Australia; 4 Department of Paediatrics, University of Melbourne, Parkville, VIC Australia; 5 Department of Biomedical Engineering, University of Melbourne, Parkville, VIC Australia; 6 Menzies Institute for Medical Research, University of Tasmania, TAS, Hobart, Australia; 7 Division of Medicine, Turku University Hospital, Turku, Finland; 8 Department of Medicine, University of Turku, Turku, Finland; 9 Heart Institute, Cincinnati Childrenʼs Hospital Medical Center, Cincinnati, OH USA; 10 Department of Pediatrics, University of Cincinnati, Cincinnati, OH USA; 11 Department of Public Health Solutions, Finnish Institute for Health and Welfare (THL), Helsinki, Finland; 12 Department of Internal Medicine, University of Turku and Turku University Hospital, Turku, Finland; 13 Department of Pediatrics, University of Colorado Anschutz Medical Campus, Aurora, CO USA; 14 Department of Epidemiology, School of Public Health, Shandong University, Jinan, China; 15 Research Centre of Applied and Preventive Cardiovascular Medicine, University of Turku, Turku, Finland; 16 Centre for Population Health Research, University of Turku and Turku University Hospital, Turku, Finland

**Keywords:** Blood pressure, Hypertension, Cardiovascular disease, Children, Adolescence, Prevention

## Abstract

**Purpose of Review:**

This review summarizes current knowledge on blood pressure in children and adolescents (youth), with a focus on primary hypertension—the most common form of elevated blood pressure in this demographic. We examine its etiology, progression, and long-term cardiovascular implications. The review covers definitions and recommendations of blood pressure classifications, recent developments in measurement, epidemiological trends, findings from observational and clinical studies, and prevention and treatment, while identifying gaps in understanding and suggesting future research directions.

**Recent Findings:**

Youth hypertension is an escalating global issue, with regional and national variations in prevalence. While the principles of blood pressure measurement have remained largely consistent, challenges in this age group include a scarcity of automated devices that have passed independent validation for accuracy and a generally limited tolerance for ambulatory blood pressure monitoring. A multifaceted interplay of factors contributes to youth hypertension, impacting long-term cardiovascular health. Recent studies, including meta-analysis and sophisticated life-course modelling, reveal an adverse link between youth and life-course blood pressure and subclinical cardiovascular outcomes later in life. New evidence now provides the strongest evidence yet linking youth blood pressure with clinical cardiovascular events in adulthood. Some clinical trials have expanded our understanding of the safety and efficacy of antihypertensive medications in youth, but this remains an area that requires additional attention, particularly regarding varied screening approaches.

**Summary:**

This review outlines the potential role of preventing and managing blood pressure in youth to reduce future cardiovascular risk. A global perspective is necessary in formulating blood pressure definitions and strategies, considering the specific needs and circumstances in low- and middle-income countries compared to high-income countries.

**Supplementary Information:**

The online version contains supplementary material available at 10.1007/s11906-024-01312-5.

## Introduction

Cardiovascular (CV) disease can trace its origins to early life [1–4], with research suggesting that blood pressure (BP) during childhood and adolescence (*youth*) can have lasting CV implications. These implications include an elevated risk of adult hypertension [5, 6], target-organ damage indicating pre/subclinical CV disease or vascular ageing [7, 8], and recent findings also point to clinical CV end-points in mid-age [9, 10]. High systolic BP (SBP) was identified as the leading risk factor for CV diseases in youths and young adults aged 15–39 years, responsible for 39.4% of the age-standardized rate of disability-adjusted-life-years [11]. These findings underscore the importance of early preventive strategies and effective interventions to manage elevated BP levels in youth. While primordial prevention is broadly supported, debate continues over the best approach for managing BP levels in youth, a contention mirrored in other CV health indicators in youth, such as cholesterol [12–14]. Central to these debates is balancing the potential benefits of early detection and management versus the potential harms of overdiagnosis, unnecessary patient anxiety, and the long-term consequences of treatment.

Initially, our goal was to update a previous review that focused on pediatric BP and adult subclinical markers of CV disease [8]. However, in compiling this update, it became clear that a broader, more comprehensive summary might benefit the field. Consequently, this review aims to provide a current overview of BP in youth, examining its classification, measurement, prevalence and trends, and potential long-term implications for CV health. We also review evidence-based recommendations for prevention and intervention, discuss promising areas of research, identify gaps in current understanding, and suggest future research directions.

## Definition of Youth Hypertension

Hypertension is categorized into primary hypertension, which arises without a clearly identifiable cause, and secondary hypertension, which results from other medical conditions affecting organs such as the kidneys, arteries, heart, or endocrine system. This review focuses on primary hypertension, with reviews on secondary hypertension available elsewhere [15, 16].

Key definitions of hypertension in youth were introduced in the 2017 American Academy of Pediatrics (AAP) [17], 2016 European Society of Hypertension (ESH) [18], and the 2004 National High Blood Pressure Education Program (referred to as the *Fourth Report*) [19] guidelines (Table 1). These guidelines defined hypertension in children as SBP or diastolic BP (DBP) greater-than or equal to the age-, sex-, and height-specific 95th percentile values. However, they differ on the ages at which adult thresholds are applied for adolescents. The Fourth Report uniformly apply these percentiles to youth aged 1–17 years [19], whereas the AAP guidelines recommend American adult cut-points (≥ 130/80 mmHg) starting from age 13 years [17], and the ESH guidelines recommend European adult cut-points (≥ 140/90 mmHg) from age 16 years [18]. Aligning hypertension definitions between adolescents and adults aims to facilitate a smoother transition in care, but use of the static thresholds, which may be above or below an adolescent’s 95th percentile, carries an inherent risk of misclassification and potentially missing early prevention and intervention opportunities. In the absence of outcome-based criteria and direct evidence on the benefits of early diagnosis and intervention, a cautious approach is justified, prioritizing patient safety and minimizing risks of overdiagnosis and overtreatment among adolescents. Nevertheless, the fixed cut-off of 130/80 mmHg for adolescents aged 13–17 years has shown to be comparable with the more complex percentile-based cut-offs in identifying those at risk [20].

**Table 1:** Classifications of blood pressure in children and adolescents in international guidelines

Guidelines	Age, years	Method	Definitions			Diagnose hypertension using repeated visits and measurements
			Normal BP	Prehypertension / high-normal /elevated BP^a^	Hypertension	
2004 Fourth Report	1 − 17	Age-, sex-, height nomograms	< 90th percentile	≥ 90th to < 95th percentile or 120/80 mmHg to < 95th percentile (whichever is lower)	Stage 1: ≥ 95th to < 99th percentile + 5 mmHg Stage 2: ≥ 99th percentile + 5 mmHg	3 visits; 3 measurements per visit
2007 The United Kingdom^b^	4 − 23	Age-, sex nomograms	< 91st percentile	≥ 91st to < 98th percentile	≥ 98th percentile	n.a
2016 ESH	≤ 15	Age-, sex-, height nomograms (same with Fourth Report)	< 90th percentile	≥ 90th to < 95th percentile	Stage 1: ≥ 95th to 99th percentile + 5 mmHg Stage 2 hypertension: > 99th percentile + 5 mmHg	3 visits; 3 measurements per visit (using the last 2)
	≥ 16	Fixed cut-offs	< 130/85 mmHg	130 − 139/85 − 89 mmHg	Stage 1: 140 − 159/90 − 99 mmHg Stage 2 hypertension: 160 − 179/100 − 109 mmHg	
2017 AAP	1 − 12	Age-, sex-, height nomograms based on normal-weight youth	< 90th percentile	≥ 90th to < 95th percentile or 120/80 mmHg to < 95th percentile (whichever is lower)	Stage 1: ≥ 95th percentile to < 95th percentile + 12 mmHg, or 130/80 to 139/89 mmHg (whichever is lower) Stage 2: ≥ 95th percentile + 12 mmHg, or ≥ 140/90 mmHg (whichever is lower)	3 visits; 3 measurements per visit
	≥ 13	Fixed cut-offs	< 120/80 mmHg	120 − 129/** < **80 mmHg	Stage 1: 130/80 to 139/89 mmHg Stage 2: ≥ 140/90 mmHg	
2018 China^b^	3 − 17	Age-, sex-, height nomograms	< 90th percentile	≥ 90th percentile to < 95th percentile; or 120/80 mmHg to < 95th percentile	Stage 1: ≥ 95th to < 99th percentile + 5 mmHg Stage 2: ≥ 99th percentile + 5 mmHg	3 visits; 2 measurements per visit (if readings differ by > 5 mmHg take 3rd measurement)
2019 JSH	Pre-schooler	Fixed cut-offs	n.a	n.a	≥ 120/70 mmHg	At least 2 visits; 2 measurements per visit (if readings differ by > 5 mmHg take 3rd measurement)
	Elementary school: first to third graders	Fixed cut-offs	n.a	n.a	≥ 130/80 mmHg	
	Elementary school: fourth to sixth graders	Fixed cut-offs	n.a	n.a	≥ 135/80 mmHg	
	Junior high school	Sex-specific fixed cut-offs	n.a	n.a	Boys: ≥ 140/85 mmHg Girls: ≥ 135/80 mmHg	
2020 HCGC	n.a	Age-, sex-, height nomograms (same with AAP)	n.a	n.a	Stage 1: ≥ 95th percentile to 95th percentile + 12 mmHg Stage 2: > 95th percentile + 12 mmHg	3 visits; 3 measurements per visit (using the last 2)
	6 − 11	Fixed cut-offs	n.a	n.a	> 120/80 mmHg	
	12 − 17	Fixed cut-offs	n.a	n.a	> 130/85 mmHg	
2022 ESC Council	0 − 15	Age-, sex-, height nomograms (same with AAP)	n.a	n.a	≥ 95th percentile	2 visits; 3 measurements per visit
	≥ 16	Fixed cut-offs	n.a	n.a	≥ 130/85 mmHg	

*AAP* American Academy of Pediatrics, *BP* blood pressure, *ESC* European Society of Cardiology, *ESH* European Society of Hypertension, *HCGC* Hypertension Canada Guideline Committee, *JSH* Japanese Society of Hypertension, *n.a.* not available

^a^In the Fourth Report guideline, the classification of “prehypertension” is termed as “high-normal” in the ESH, United Kingdom, Canada and China guidelines, and “elevated” in the AAP guideline

^b^Blood pressure reference values derived from their own youth. Other guidelines used reference values from US youth

The AAP, ESH, and Fourth Report all use normative BP values derived from United States (US) youth to establish age-, sex-, and height-specific BP percentiles. However, they differ in their inclusion criteria. The Fourth Report includes data from youth across all weight categories [19], whereas the AAP uses data only from normal-weight youth [17]. The ESH guidelines initially endorsed values from the Fourth Report [18], but the latest consensus advised shifting to the AAP’s tables for youth under 16 years [21]. The rationale for the modified BP tables is that normative BP values derived from data including youth with overweight and obesity push cut-offs to higher BP levels compared to those restricted to normal-weight youth, potentially misclassifying BP status [17].

Outside of the US, countries like China [22], India [23], and the United Kingdom (UK) [24] have established national normative BP data for youth reflecting the local demographic (summarized in Table 1). Moreover, international age-, sex-, and height-specific BP references have been constructed for youth aged 6 to 17 years across seven countries [25], although no such data exist for African youth [26]. Some guidelines, such as Hypertension Canada Guideline Committee [27], the Japanese Society of Hypertension [28], and a consensus from the European Society of Cardiology [21] recommend fixed cut-offs for some or all age-groups of children and adolescents (Table 1).

The rationale for using these fixed, and inherently *simplified*, cut-offs is evidence from the Bogalusa Heart Study that BP classified using fixed cut-offs (children aged 6–11 years: SBP/DBP ≥ 120/80 mmHg; adolescents aged 12–17 years: SBP/DBP ≥ 130/85 mmHg) associated with increased risk of adult hypertension and subclinical CV damage [29]. These fixed cut-offs also cross-sectionally associated with childhood markers of vascular structure and arterial stiffness in an international consortium [30, 31]. Interestingly, these two fixed cut-offs had the same utility as the percentile-based definition from the Fourth Report that encompasses up to 238 cut-offs for the 95th percentiles of BP (17 ages*2 sexes*7 height percentiles) [29], and those from the AAP guideline [30, 31]. Further, in a large sample of youth from seven countries, the height-specific 95th BP percentile values, with 22 fixed cut-offs (11 height categories in increments of 10-cm from 80 to 180 cm*2 sexes), performed best among 11 simplified methods, including the abovementioned two fixed cut-offs, having a higher positive predictive value (PPV) of ~ 90% and negative predictive value (NPV) of 100% compared with the complex cut-offs in the Fourth Report [32].

Despite the variation in hypertension definitions [17–19, 21, 22, 27, 28], there is consensus on the need for multiple BP measurements across repeated visits to confirm diagnosis. However, differences exist regarding the number of required visits (two or three) and measurements at each visit (two or three), and whether the first measurement is included in the diagnostic assessment (Table 1). These requirements pose challenges in practice, including increased healthcare costs and barriers to patient compliance [33], underscoring the need for practical and balanced approaches in clinical practice.

## Blood Presure Measurement in Youth

Best practice principles for BP measurement in youth are relatively consistent across international guidelines and share similarities to those for adults. A key issue is the limited number of automated devices that have passed independent validation for accuracy in youth [34]. Currently, www.stridebp.org lists only 14 office/clinic devices and 3 devices each for home and ambulatory use. While current guidelines allow for initial screening with automated devices, confirmation of apparently high BP with the auscultatory method is recommended given that the first and fifth Korotkoff sounds were used to develop normative tables [17, 18, 27].

White coat hypertension (hypertensive office BP but normal out-of-office BP) affects 4–10% of youth in the community [35, 36], and 13–46% of children referred to specialist clinics for suspected hypertension [37–39]. Thus, ambulatory blood pressure monitoring (ABPM) is required to confirm a hypertension diagnosis, though its use is limited to children that are old enough to tolerate it. Although the AAP guidelines mention an approximate minimum age of 5 years, poor tolerability was reported in 32% of 14–16-year-olds, highlighting the need for technological development in pediatric ABPM [40]. The need of more comprehensive normative ABPM data—covering a broader geographical area and demographic range, and use of widely-used devices—is also recognized [41]. ABPM can also detect masked hypertension (normal office BP but hypertensive out-of-office BP), which occurs in 10.4% of youth and is associated with subclinical CV outcomes including left ventricular hypertrophy and higher pulse wave velocity (PWV) [42, 43].

While population-wide screening for masked hypertension may not be feasible, ABPM is recommended to identify masked hypertension and abnormal circadian BP variations in high-risk youth, including those with chronic kidney disease, diabetes, obesity, sleep-disordered breathing, or a history of aortic coarctation [41]. Particularly for those being treated for hypertension, there is evidence supporting the supplemental use of home BP monitoring alongside ABPM [44]. However, implementing home BP monitoring presents several obstacles, including the accuracy of unvalidated devices and practical challenges related to home measurement. Cultural factors, along with access and affordability issues, further complicate the adoption of both ABPM and home monitoring in some countries and settings [26].

## Prevalence and Trends of Hypertension in Youth

An older systematic review and meta-analysis of 47 studies from 21 high-income and 26 low- and middle-income countries found the global prevalence of hypertension in those under 19 to be 4.00% [95% confidence interval (CI), 3.29, 4.78)], with higher prevalence in low- and middle-income countries [4.43% (3.16, 5.90)] compared to high-income countries [3.52% (2.74, 4.39)] [45]. Although this study used BP measurements obtained on at least three separate occasions, it used the Fourth Report instead of the AAP definitions, which may underestimate the prevalence. There has been a global rise in the prevalence of youth hypertension in recent decades: 1.26% (0.79, 1.84) in the 1990s, 3.30% (2.69, 3.97) in the 2000s, and 6.02% (4.38, 7.91) from 2010–14 [45]. However, trends vary by region.

In the US, after two-decades of decline, youth hypertension has plateaued or slightly increased according to AAP guidelines. Prevalence among children aged 8–12 years was 5.2% in 1999–2002, peaked at 6.2% in 2003–2006 and then decreased to 5.0% in 2007–10, with a slight further decline to 4.7% in 2011–14 and then stabilizing to 4.6% by 2015–18 [46]. Over the same period, adolescents aged 13–17 years had a steady decrease: 6.6% in 1999–2002, 5.5% in 2003–06, 4.6% in 2007–10, then bottomed out at 2.5% in 2011–14, before increasing to 3.7% in 2015–18 [46]. In China, youth aged 1–20 years had a consistent decline in hypertension prevalence (based on ≥ 95th percentile, though specific norms were not disclosed) from 18% in 2007 to 11% in 2010, and a further decline to 6% in 2014 [47]. In Korea, using the AAP guidelines, hypertension prevalence increased in youth aged 10–18 years from 5.4% in 2007–11 to 7.8% in 2012–16 and further to 9.9% in 2017–20 [48]. Similarly, in India, youth aged 1–18 years showed a rising trend in hypertension (defined as ≥ 95th percentile, with the BP norms not reported) before declining: 2% before 2000, 3% from 2001–05, 9% from 2006–10, 7% from 2011–15, and 6% from 2016–20 [49]. In Africa, data from meta-analysis suggests an upward trend with studies from 1996–2017 estimating a 5.5% prevalence of hypertension using the Fourth Report definition [50], while studies from 2017–20 estimate the prevalence at 7.5% according to the AAP guideline [51].

When evaluating prevalence estimates, it is important to recognize the deviation from guideline-recommended diagnosis of hypertension, which require consistently elevated BP across multiple measurements and clinic visits, and the single-visit measurements predominantly used in these prevalence studies. This practice likely leads to an overestimation of hypertension prevalence in youth. However, provided the definition of hypertension and the measurement methods are consistent, the observed trends should reflect true changes in prevalence.

## Key Risk Factors for Pediatric Hypertenion

The global rise in pediatric hypertension is primarily attributed to the coinciding obesity epidemic among youth [33], but this trend is not universal. For example, China [52] has reported a decline in hypertension prevalence despite rising obesity, suggesting that multiple factors contribute to pediatric hypertension. According to the AAP guideline, these factors include older age (> 6 years) prenatal and maternal risk factors (maternal hypertension, low birth weight), lifestyle factors such as physical inactivity and poor nutrition (high salt intake, low fruit and vegetable consumption), and negative youth/prenatal experiences (maltreatment, anxiety) [17]. Other contributing factors are sleep disorders (sleep duration ≤ 8 h/night, poor sleep quality, sleep deprivation, obstructive sleep apnea) [53–55], sedentary behaviors (screen time > 2 h/day) [56], tobacco exposure (both active and passive smoking) [57], environmental factors (air pollution) [58], and social determinants (low family income) [46]. Additionally, sex and race differences play a role, with males and Blacks showing higher BP levels from the age of 12 years [59].

Familial aggregation or resemblance of BP shows that hypertension traits cluster within families due to genetic and shared environmental factors [60]. The HERITAGE Family Study reported maximal heritability (genetic and environmental) of 46% for SBP and 31% for DBP, with this higher among Blacks (SBP, 68%; DBP, 56%) than Whites (SBP, 43%; DBP, 24%) [61]. Approximately 50–60% of BP familial aggregation is genetic heritability [62], particularly in early-onset hypertension [63]. Together, familial aggregation studies outline the potential to identify hypertension and manage risk early in life.

Family history is a valuable predictive tool in pediatric settings, where children of hypertensive parents have higher and faster rising BP levels from infancy [64, 65]. Niiranen et al. [63] found that adults whose parents were diagnosed with hypertension at a younger age had higher risk of hypertension themselves. However, parental hypertension history does not account for the full genetic risk as it includes non-genetic heritability factors such as shared behaviors and environments. Adding parental hypertension history with youth BP and modifiable risk factors improves prediction of adult hypertension, but including genetic risk can further refine these predictions [66].

Genome-wide association studies identify genetic variants associated with elevated BP [67, 68] that can be compiled into a genetic risk score (GRS) or a more extensive polygenic risk score (PRS). While both scores predict genetic predisposition to hypertension, the PRS covers a broader range of genetic influences, and its use is becoming more widespread due to advances in computational and genomic technologies. In the Cardiovascular Risk in Young Finns Study, a GRS derived from 13 SNPs associated with life-course BP since childhood [β for 1-standard deviation (SD) increase in GRS: 0.47 mmHg for SBP; 0.53 mmHg for DBP, all P < 0.01, 95% CI not reported], even after adjusting for age, sex, and body mass index [69]. Similarly, pooled data from two studies found a GRS based on 29 SNPs associated with SBP at age 6 years [per-allele effect sizes, β (standard error) 0.097 (0.039) mmHg], but not with the changes between ages 6 to 17 years [70]. The genetic determinants of BP may evolve as individuals age [71], indicating variability in the genetic drivers of hypertension at different life stages. A recent initiative developed a youth-specific GRS for hypertension including 16 SNPs with the aim to enhance the genetic foundations of hypertension in youth and aid prediction [72]. However, translating these genetic insights into clinical practice faces significant challenges and requires further research to identify barriers and potential solutions.

Ultimately, hypertension in the young arises from a complex interplay of lifestyle, environmental, and genetic factors. The extent to which lifestyle modifications can mitigate the impact of genetic predispositions on BP across the life-course remains unclear. However, a study on adults suggests that lifestyle impacts on incident hypertension are consistent across different PRS groups [110].

## Consequences of Youth Blood Pressure

Elevated BP in youth increases the risk of adult hypertension and is adversely associated with both immediate and long-term cardiovascular health outcomes. This includes vascular aging (characterized by structural and functional alterations in the vasculature) and cardiac damage, and overt CV disease in adulthood.

### Youth Blood Pressure and Adulthood Blood Pressure

Substantial data published from cohort studies over the last 50 years show the tendency for BP to track (or persist) from youth to adulthood; summarized in meta-analyses by Chen et al. (50 studies, correlation coefficients of 0.38 for SBP and 0.28 for DBP) and Toschke et al. (29 studies, correlation coefficients of 0.37 for SBP and 0.31 for DBP) [5, 6]. Recently, an international study involving 38,589 participants found a correlation coefficient of 0.44 for SBP tracking from childhood to mid-adulthood over 35 years [9], while a meta-analysis showed that each 1-SD increase in youth SBP increases the odds of adulthood hypertension by 1.72 times (95% CI, 1.50, 1.95) [73]. A summary of key studies that assess the potential of raised BP in childhood to predict adult hypertension is provided in Table S1. However, these studies tend to have small sample sizes, lack, or do not consider, BP measurements in the intervening period, or use non-standardized definitions for high-risk BP categories. As such, the US Preventive Services Task Force (USPSTF) has called for future research to adopt standardized definitions [74], such as the AAP-defined high thresholds [75], to enhance understanding of hypertension transition from youth over the life-course.

Recent cohort data has also examined BP developmental trajectories, using multiple measurements that span youth through to adulthood. From puberty, males exhibit a steeper increase in SBP, peaking in young adulthood (age 26 years) at levels 8.2 mmHg (95% CI, 6.7, 9.8) higher than in females [76]. Females tend to catch up by their seventies, having more rapid increases in SBP later in life [76]. Data from the Bogalusa Heart Study supports these findings, showing distinct SBP and DBP trajectories by sex and race from 4 to 51 years [59]. This study also found higher BP levels and rates of increase throughout puberty and young adulthood associated with increased odds of hypertension in mid-adulthood [59]. Despite debate over the clinical application of these trajectory analyses [77], they, along with the tracking data, suggest the early origins of adult hypertension begin in youth, providing opportunities for early prevention.

### Youth Blood Pressure and Target-Organ Damage

Cross-sectional studies (detailed in Table S2) have shown the association between youth BP and target-organ injury including vascular alterations such as increased carotid intima-media thickness (IMT) and arterial stiffness, cardiac changes indicated by higher left ventricular (LV) mass index (LVMI), and potential cognitive impairments. However, the association between youth BP and kidney injury, particularly microalbuminuria (urine-albumin-to-creatinine-ratio), remains unclear with current data not showing significant differences between normotensive and hypertensive adolescents [78], but there is a need for more sensitive markers to detect early kidney injury.

The long-term association of youth BP with markers of target-organ damage in adulthood (Table S3), focusing mainly on structural [IMT, coronary artery calcification (CAC)], mechanical [arterial stiffness, e.g., PWV, carotid distensibility (cD)], and functional [brachial flow-mediated dilatation (FMD)] markers of subclinical CV disease, and markers of cardiac damage (LVMI) [79–99] is well established. However, limited evidence exists for outcomes of cognitive function and kidney health [100–102]. An important systematic review and meta-analysis of 12 cohorts published in 2020 found that elevated BP in childhood accelerates vascular aging [high PWV (defined as ≥ age-, sex-, and heart rate-/study-specific 80th percentiles): OR (95% CI), 1.83 (1.39, 2.40); high carotid IMT (defined as ≥ age-, sex-, and study-specific 90th percentile): 1.60 (1.29, 2.00)], and increased the odds of developing LV hypertrophy (defined as LVMI > 49.2 g/m^2.7^ in males and LVMI > 46.7 g/m^2.7^ in females) [1.40 (1.20, 1.64)] in adulthood [7]. Moreover, findings from the i3C Consortium suggest youth SBP thresholds predictive of increased adult carotid IMT are lower than current definitions for elevated youth BP (90th percentile) by the AAP [88]. These insights call for reevaluation of existing definitions to better align with preventive efforts [103].

### Life-Course Blood Pressure and Target-Organ Damage

Recognizing the role of youth BP on adulthood subclinical CV risk, research has begun to address risk development across the life-course from childhood to adulthood. Studies using BP data from two time-points have shown that individuals with persistently elevated BP in both youth and adulthood have the highest risk for adult markers of early vascular aging (carotid IMT [104] and PWV [105]). Conversely, those who resolve their elevated BP from childhood to adulthood largely do not show an increased risk, although a small residual risk remains. These results provide impetus for outlining modifiable factors associated with the resolution of raised BP [106] that could be targeted across the life-course.

However, analyses using BP from only two time-points in youth and adulthood could not fully assess the impact of the duration or timing of exposure [104, 105]. Research involving multiple BP measurements from youth to adulthood that consider cumulative BP (usually calculated as the area under the curve from serial measurements [89, 107–110] or from trajectories [111, 112]) suggests that prolonged exposure to high BP contributes to vascular aging and cardiac damage. A recent study outlined cumulative BP as the most important risk factor for markers of early vascular aging (PWV, IMT) compared with other factors such as lipids and body mass index [107]. While useful in describing long-term BP burden, these analyses do not determine specific life stages at which the BP-outcome association may vary—information that could optimize screening and prevention efforts.

In response, recent studies have employed advanced life-course modelling to determine the relative contributions of BP at different life-stages. These studies consistently find that while BP at each examined life-stage directly associates with the outcomes, the extent of the contribution varies depending on the outcome. For example, BP at different life-stages from infancy appears to provide an equal contribution to adult carotid IMT [113], whereas for PWV, BP levels measured closest to the outcome contribute most [114]. These findings suggest that while maintaining lower BP levels throughout life is ideal, controlling high BP at specific life-stages can still likely reduce CV risk.

Despite the value of these studies in understanding the long-term risks associated with BP, the complexity of the required data limits their practical application in clinical settings. An alternate, more feasible approach for clinicians might involve using the age at hypertension onset both in parents [63] and in the child to assess risk, with earlier onset related to higher risk of incident subclinical CV disease [115]. However, this research has primarily been conducted in adults, leaving a gap in our understanding of how knowledge of early versus later onset hypertension affects youth, noting our discussion above on tracking of BP from youth to adulthood.

### Youth Blood Pressure and Clinical Cardiovascular Disease

While there is substantial evidence linking youth BP with subclinical CV risk, its association with clinical CV events is less well established. Early studies, like the Swedish 1969 male conscript cohort first indicated an association between young adult (aged 18–20 years) BP and incident coronary heart disease [116], and stroke [117]. However, these results contrasted with those from younger participants (aged 6–18 years at baseline) in the Princeton-Lipid Research Clinics Follow-Up Study where elevated youth BP (≥ 90th percentile using the Fourth Report) did not associate with incident CV disease 26 years later [118].

The link between youth BP and CV mortality in adulthood was initially highlighted in the Harvard Alumni Health Study among 855 males aged 19 years at baseline with a mean follow-up of 30 years [119]. These findings were confirmed in a subsequent study with a longer follow-up duration and lager sample size that accounted for the potential influence of adult BP [120]. Comparable conclusions were drawn from an Israeli cohort where youth (aged 16–19 years) hypertension (SBP/DBP ≥ 140/90 mmHg) was associated with stroke mortality [121]. Another study among Swedish male conscripts found that among young adults (mean age 18.4 years), DBP above 90 mmHg was positively associated with all-cause mortality over a median follow-up of 24 years whereas SBP demonstrated a U-shaped relationship with mortality risk, showing the lowest risk at a level of 130 mmHg [122]. This U-shaped curve might be explained by associations of low SBP with factors such as poor social, physical, and mental wellbeing, along with issues in brain perfusion and reflex abnormalities. The study also addressed concerns of reverse causation, where pre-existing illness might lead to both low BP and higher mortality. Such concerns are minimized by the young age and low prevalence of disease of the cohort at baseline, and the findings remaining consistent even after excluding data from the early years of follow-up. Another study failed to show the association of hypertension (using the cut-offs from the Fourth Report) in younger American Indian youth (aged 5–19 years) and all-cause mortality over a median follow-up of 23.9 years [123]. These varied findings underscore the complex relationship between youth BP and adult CV events, highlighting the need for further investigation. Important insights have been gained from recent studies.

The i3C Consortium found youth SBP associated with increased risk of both fatal [per 1-SD, HR (95% CI), 1.34 (1.19, 1.50)] and fatal/nonfatal [1.33 (1.24, 1.42)] CV events in adulthood [9]. This study also found that SBP levels in the 50th to 90th percentiles—considered normal by most current guidelines—associated with increased risk, which was consistent with earlier research on subclinical CV disease. Follow-up of almost 2 million Israeli military conscripts recently showed that those with hypertension in adolescence had approximately twice the risk of overall and ischemic stroke at a median age of 43 years compared to those without hypertension [10]. While these studies have typically defined hypertension among generally healthy individuals, a recent study identified youth with more severe or symptomatic hypertension using hospitalization and physician claims, including both primary and secondary causes. This study found that youth with hypertension had a 2.06-fold higher risk of major adverse cardiovascular events over a median follow-up of 12.9 years compared to those without hypertension (95% CI, 1.94–2.19) [124].

Recent evidence from the Coronary Artery Risk Development in Young Adults (CARDIA) Study found that 15-year cumulative SBP from young adulthood (aged 18–30 years), quantified as area under the curve, associated with increased risk for heart failure [per 1-SD increase: HR (95% CI), 2.14 (1.58, 2.90)], coronary heart disease [1.49 (1.19, 1.87)], and stroke [1.81 (1.38, 2.37)] [125]. Mendelian randomization analyses in the UK Biobank have further confirmed that genetically lower SBP across the life-course (2.9 mmHg lower on average) associated with 18% (95% CI, 0.79, 0.85) reduced odds of CV events [126]. Although this analysis did not specifically involve youth data.

Collectively, these studies outline the potential immediate and long-term CV benefits of early and effective BP management. This evidence needs to be considered with the generally lower awareness, diagnosis, and management of hypertension in younger populations compared to older adults, which might be further amplified in those with disadvantage [127]. The next section provides an overview of approaches for the prevention and treatment of elevated BP in youth.

## Prevention and Treatment

### Therapeutic Lifestyle Intervention

BP guidelines consistently highlight the fundamental role of lifestyle modification to prevent and treat abnormal BP in youth. [17, 18, 27] This is supported by several short-term randomized trials (refer to Supplement Text 1 for key trials) where lifestyle interventions aimed at lowering salt intake [128], promoting healthier diet choices [64] or regular physical activity and exercise [129] (especially high-intensity interval-based training [130]), and losing weight (reduction in body mass index standard deviation score) [131] have proven effective in lowering youth BP. Given the multifactorial nature of BP, the design of future lifestyle interventions should consider more detailed aspects of diet (microbiome [132]), mental health (symptoms of anxiety, depression and stress) [133], and the 24-h activity cycle (ideal time allocation) [134]. Furthermore, policies to control pollutants and environmental chemicals including outdoor (fine particles in ambient air) and indoor (household air pollution from inefficient household combustion) air pollution, environmental toxicants (heavy metals), noise pollution, and passive smoking, especially in low- and middle-income countries, seem pertinent [135].

### Pharmacological Intervention

The decision to initiate pharmacological treatment for hypertension in youth depends on several factors (detailed in Table S4) [17, 18]. The safety and efficacy of antihypertensive medicine is less well-documented in youth compared to adults. A review by Gartlehner et al. which included six clinical trials with 909 participants aged 6 to 17 years, found that the risk of adverse events (such as dizziness and headache) was comparable between pharmacologic treatment and placebo over 2 to 4 weeks [74]. In terms of efficacy, a network meta-analysis involving 2,378 hypertensive children of median age of 12 years across 13 randomized placebo-controlled clinical trials (median follow-up of 35 days) found that angiotensin converting enzyme inhibitors and angiotensin receptor blockers, but not calcium channel blockers and thiazide diuretics, significantly reduced SBP and DBP compared to placebo [136]. Although these four drug classes are recommended as first-line treatments [17], individual responses vary [137], suggesting the need for personalized treatment plans. Data have emerged demonstrating that angiotensin converting enzyme inhibitors can lead to regression of LV hypertrophy (the process of reducing LVMI) [138], but similar evidence is lacking for other drugs, and there is no direct evidence linking LV hypertrophy reversal in youth with reduced CV risk later in life.

Overall, while clinical trials demonstrate short-term efficacy and safety of antihypertensive treatments in youth, evidence of their long-term efficacy and safety remains limited. This lack of long-term data contributes to the discordance and variability in guidelines and recommendation statements regarding BP screening and treatment in youth. The next section summarizes the various BP guidelines and recommendation statements for youth.

## Screening in Youth: No Consensus

There is a lack of long-term randomized controlled trials that quantify the benefits and harms of BP screening and management in youth, particularly evidence demonstrating that early screening for abnormal BP can reduce the risk of future CV events. Reflecting this gap in evidence, the USPSTF in 2003 concluded that the evidence was insufficient to recommend for or against routine BP screening in youth [139]. Subsequent updates [140, 141] reaffirmed this stance, indicating an ongoing uncertainty in assessing the *balance of benefits and harms of screening in children and adolescents*. In contrast, guidelines from the AAP [17], the ESH [18], and the Hypertension Canada Guideline Committee [27] recommend BP screening in youth. Table 2 summarizes the recommendations for BP screening from these guidelines with the following section overviewing the evidence and challenges associated with BP screening in youth.

**Table 2:** Recommendations on screening of blood pressure in youth

Guidelines	Evidence quality	BP screening recommendations	Screening methods			
			Devices	Number of readings at a single visit	Time interval between consecutive readings at a single visit	Confirm hypertension within separate visits
2016 ESH	n.a	Initiate at age ≥ 3 years; Repeat if BP is normal	Preferred: Auscultatory; Confirmatory: ABPM	≥ 3, use the average of the last 2	3 min	≥ 3 visits in 1 year
2017 AAP	Moderate	Annual for age ≥ 3 years; History of obesity, renal disease, diabetes, or aortic arch coarctation: Measure BP at each health care encounter	Oscillometric confirmed by auscultatory Confirmatory: ABPM	If the initial BP is elevated (≥ 90th percentile), take 2 additional measurements	n.a	Elevated BP: Repeat on 2 additional visits in 6 months intervals Stage 1 hypertension: Repeat on 2 additional visits in 1–2 week to 3 month intervals Stage 2 hypertension: Repeated on 1 additional visit within 1 week
2020 HCGC	Expert opinion	Regularly measure from age ≥ 3 years	Preferred: Auscultatory; Confirmatory: ABPM	≥ 3, use the average of the last 2	n.a	Stage 1 hypertension: Repeat on ≥ 2 occasions within 1 month Stage 2 hypertension: Prompt referral
2020 USPSTF	Insufficient evidence	Evidence lacking for benefits/harms balance				

*AAP* American Academy of Pediatrics, *ABPM* ambulatory blood pressure monitoring, *BP* blood pressure, *ESH* European Society of Hypertension, *HCGC* Hypertension Canada Guideline Committee, *n.a.* not available, *USPSTF* United States Preventive Services Task Force

### Potential Efficacy and Harms

High quality clinical trials for childhood BP screening and intervention to prevent adult events might never be possible and would be complex, expensive and require a large sample size. One of the arguments for screening is that we should not justify inaction (in the presence of so much other evidence) in the absence of potentially unobtainable evidence that some authorities require [142]. For example, cohort studies simulate efficacy by showing those able to resolve elevated youth BP have reduced odds of developing hypertension and markers of early vascular aging (carotid IMT [104] and PWV [105]) in adulthood, though data on CV endpoints is lacking. Additionally, findings from cross-sectional and longitudinal studies reinforce the potential benefits of early screening (Tables S2 and S3). Moreover, the primary physiological mechanism in pediatric hypertension is increased circulating volume, leading to elevated and sustained peripheral vascular resistance—a precursor to adult hypertension [143]. Measuring BP in pediatric physical examinations was advocated in the National Institutes of Health-sponsored expert panel report [144], and endorsed in the 2017 AAP guideline [17], emphasizing its importance in the early detection and management of hypertension. The implementation of BP measurements in routine school entry physical exams in some regions, such as in the US [145], exemplifies the feasibility of early hypertension screening.

The potential harms relate to high false-positive hypertension diagnosis in youth, including overdiagnosis and unnecessary treatment. Accurate BP evaluation is a basis of screening, yet guidelines lack consensus on measurement protocols (Table 2). Recent evidence underscores the need for consistency among pediatric guidelines in recommending repeat BP measurements for hypertension diagnosis, as there is considerable individual variation in SBP change among consecutive readings at each single visit that can influence BP classification [146]. ABPM improves the accuracy of BP evaluation by capturing day and night fluctuations [17], but multiple BP readings could increase the chances of diagnosing hypertension. Furthermore, the potential discomfort caused by ABPM during sleep can contribute to an overestimation of ambulatory hypertension, underscoring the importance of educating patients about the procedure. Additionally, ABPM-specific hypertension thresholds are understudied. The optimal balance between accurate evaluation versus feasibility and cost of screening, especially in low- and middle-income countries with limited resources, remains under examined. Weak predictive ability from youth BP to future CV risk is another concern (C statistics≈0.60–0.68 for adulthood hypertension, carotid IMT and PWV) [147], although screening protocols with adjunctive predictors such as familial predisposition, obesity, and genetic data might improve prediction [66].

### Challenges in Hypertension Diagnosis

The first challenge relates to youth hypertension thresholds. The current threshold for youth hypertension set at the 95th percentile was criticized by the USPSTF as an “arbitrary statistical point” [144]. Using this hypertension threshold to predict adult CV risk results in high specificity but low sensitivity, suggesting some at-risk individuals remain undetected [118]. Recent evidence suggests employing a lower percentile (70th-80th percentile for SBP) for screening to increase sensitivity while minimizing reduction in specificity, improving early identification without significantly increasing false positives [147]. In support of revised hypertension definitions, mentioned above, evidence is consistently showing subclinical/clinical CV risk can begin at BP levels below the conventional 95th percentile threshold [9, 88]. Moreover, the proportion of adult elevated BP attributable to elevated child BP was estimated to only be about 10% [106], and youth with BP elevations within the normal range can still progress to adulthood hypertension [147], highlighting the benefits of early prevention. The AAP guidelines respond to these findings by recommending more inclusive cut-offs for initial screening [17]. Consensus is emerging that lowering youth BP thresholds is warranted [103, 148]. However, lowering thresholds will need to be weighed against the added burden—due to higher prevalence of hypertension—on the healthcare system this will create, and will vary by country/region.

The complexity inherent in pediatric BP classifications—owing to their reliance on a matrix of age-, sex-, and height-specific percentiles—poses another significant challenge for routine screening. The AAP guidelines provided simplified cut-offs for initial screening for children aged 1–12 years, based on the sex- and age-specific 90th BP percentile value at the 5th height percentile [17]. However, this approach showed low PPV of < 50% [149]. In contrast, data from the NHANES showed a simplified table using a higher height percentile (75th) had both high PPV (85.0%) and negative predictive values (NPV) (98.6%) in children aged < 13 years [150]. Additionally, the 95th BP percentile with 22 fixed cut-offs emerged as the most effective among 11 simplified options, offering the highest PPV (~ 90%) and a NPV of 100%, outperforming the more complex Fourth Report cut-offs [32]. However, the age restriction above six years and the lack of CV endpoints in these studies, and the potential impact of variation by population factors needs to be determined [149].

### Cost-Effectiveness of Blood Pressure Screening

Cost-effectiveness of any screening program is a central consideration. A simulation study in the US population aged ≥ 18 years showed that population-based screening of BP is modestly cost-effective (USD 48,500/quality-adjusted life year) [151]. In another simulation study among US adolescents aged 15 years, neither population-based screening coupled with treatment for hypertension and LV hypertrophy, or targeted screening among adolescents with overweight/obesity, exhibited superior cost-effectiveness than population-level lifestyle modifications (salt reduction, increased physical activity) [152]. However, these studies did not consider younger age groups, other possible subgroups being targeted (e.g., with family history of hypertension), frequency of screening, cost of personnel, subsequent treatment costs, and likely lack of generalizability to other countries and health systems. The application of machine learning might assist in disentangling these uncertainties [153].

### Blood Pressure Screening in Low- And Middle-Income Countries

Guidelines on youth BP screening have only been established in high-income countries. However, screening strategies in low- and middle-income countries require particular attention due to the challenges these regions face, including other essential health needs with limited healthcare resources, disparities in health literacy, and higher hypertension prevalence [135]. Adult studies in developing countries have shown community-based screening (at schools and community events) involving task-shifting to non-physicians and community-based health workers as an effective approach to screen and manage BP [154, 155]. Digital health technology (e.g., education program with text messages, using mobile devices and apps) is another promising strategy in locations where these devices are pervasive [156], as it allows for remote monitoring and can improve access to healthcare services in rural or hard-to-reach areas.

## Conclusion

We positioned recent research on BP in children and adolescents within the broader field. We advocate for attention on the prevention and management of BP in youth, maintaining a cautiously optimistic standpoint. While we recognize primordial prevention as the ideal, early screening and subsequent interventions hold promise. We outline needs for ongoing research to continue to address the critical evidence gaps (Table 3) and advocate for a global perspective to formulate BP definitions and prevention strategies tailored to the unique contexts of low- and middle-income versus high-income countries.

**Table 3:** Research gaps in pediatric hypertension

Research gaps	Potential opportunities
*Measurement: office BP*	
Disagreement on office BP measurement protocol: the number of readings and whether to discard the first reading at each visit, the required number of visits for diagnosis, and the time interval between consecutive visits.	Conduct clinical trials comparing different BP measurement protocols regarding their ability to predict cardiovascular outcomes. Leverage data from existing longitudinal cohort studies to compare the predictive utility of hypertension, diagnosed using various BP measurement protocols, on future cardiovascular risk. Evaluate the cost-effectiveness of different BP measurement protocols.
Normative BP values largely limited to youth in the United States.	Invest in large-scale epidemiological research to establish BP norms for various racial and ethnic groups.
*Measurement: Ambulatory BP monitoring*	
Accuracy and predictive utility of ABPM in youth, especially those with obesity.	Conduct studies to validate the accuracy of existing and new automated BP devices in diverse pediatric populations. Compare predictive utility of youth BP from automated devices versus office measurements to subclinical/clinical cardiovascular risk.
Normative data, especially among youths aged less than 5 years and of smaller stature, and from more diverse populations.	Conduct nation-wide studies to gather ambulatory BP monitoring data across wider age and height groups, as well as various ethnic, geographical, and socioeconomic groups. Then, establish age-, sex-, and height-specific reference standards.
Number of ambulatory BP readings needed for accurate hypertension diagnosis in youth or best future subclinical/clinical cardiovascular disease prediction.	Compare the diagnostic accuracy of hypertension in youth using a varying number of ambulatory BP readings. Determine the ideal number of ambulatory BP readings in youth for long-term cardiovascular health prediction.
*Definitions of hypertension*	
Youth hypertension definitions (based on age-, sex-, height- percentile) are associated with end organ damage, however have not been related to cardiovascular events.	Determine the association of discrete thresholds of systolic and diastolic BP in youth with risk of fatal and non-fatal events in adulthood. Evaluate if simplified thresholds have similar associations with events compared with percentile-based thresholds.
*Hemodynamics of primary hypertension*	
Limited understanding of how primarily systolic hypertension in youth (due to increased cardiac output) transitions to predominantly diastolic hypertension in middle age (reflecting increased systemic vascular resistance).	Conduct longitudinal studies to track the evolution of hypertension from youth to middle age, focusing on the transition from increased cardiac output to increased systemic vascular resistance. Investigate the underlying mechanisms and risk factors contributing to this phenotypic shift in hypertension. Assess the effectiveness of different treatment strategies tailored to the changing nature of hypertension across the life-course.
*Risk factors*	
Limited data on the mechanisms driving hypertension development from a very early age and its long-term implications for cardiovascular health.	Examine the impact of early-life factors (such as nutrition from infancy) on hypertension trajectories. Clarify the roles of genetic predispositions and epigenetic modifications in the development of hypertension in very early life and their impact on cardiovascular risk later in life. Explore the interplay between youth hypertension, obesity, and metabolic syndrome, and discern the impact of BP on cardiovascular risk in the absence of the other two factors.
Association of parental hypertension onset timing (early vs. later) on the risk of hypertension in youth offspring.	Implement studies that measure BP across multiple generations. These studies should systematically track both BP and health behaviors within families over time, noting the age at which parents were diagnosed with hypertension and its correlation with hypertension onset in their children. Using genome-wide and epigenome-wide association studies to identify the genetic and epigenetic markers associated with early vs. later onset hypertension in parents. Analyze how these factors influence the risk of hypertension in offspring. Study the effectiveness of lifestyle interventions in individuals with a family history of early-onset hypertension.
Clinical utility of genetic information in pediatric hypertension management is understudied.	Investigate how genetic variants interact with environmental and lifestyle factors in the development of hypertension. Understand how genetic information can guide treatment choices for pediatric hypertension. For example, compare the responses of patients to treatment based on different genetic information. Evaluate the cost-effectiveness and accessibility of genetic testing. Consider different phenotyping (hypertension classification, ambulatory BP and BP trajectories) in combination with genetic information.
*Target-organ injury*	
Assess adverse renal changes related to youth hypertension.	In epidemiological studies, determine the prevalence and patterns of renal changes in hypertensive youth. Track renal changes in youth with hypertension over time to understand when and how traditional markers like microalbuminuria become relevant. Investigate mechanisms of hypertension mediated renal damage in youth and understand how this compares to adults. Validate non-invasive imaging techniques (e.g., magnetic resonance angiography) and biomarkers (e.g., genomic and proteomic markers) that are sensitive to renal changes in hypertensive youth.
*Prevention and intervention*	
No direct evidence from clinical trials that examine if screening for hypertension in youth at all ages is effective in reducing future cardiovascular risk.	In the absence of long-term clinical trials, use observational data from longitudinal cohorts: • To examine the associations among childhood hypertension, adult hypertension, and cardiovascular endpoints in adulthood. • To investigate the natural transition (resolution and progression) of hypertension from youth to adulthood and investigate how these changes are associated with future cardiovascular endpoints.
Limited information for establishing goals for lifestyle interventions	Clinical trials to • Determine the amount of weight loss required to achieve clinically meaningful reductions in BP, particularly among youth with obesity. • Investigate the interaction between lifestyle modifications and genetic markers. This could lead to more personalized BP targets (precise medicine).
Limited information on pharmacological treatment efficacy, priorities, and decisions.	Clinical trials to: • Compare the efficacy and harms of different types of drugs and investigate the efficacy of combination medications. • Determine if drug efficacy differs by the absence and presence of preclinical cardiovascular damage. • Explore how genetic variations affect responses to antihypertensive medications in children.

*ABPM* ambulatory blood pressure monitoring *BP* blood pressure

## Supplementary Information

Below is the link to the electronic supplementary material.

Supplementary file1 (DOCX 168 KB)

## Data Availability

No datasets were generated or analysed during the current study.

## Abbreviations

AAP: American Academy of Pediatrics
ABPM: Ambulatory blood pressure monitoring
BP: Blood pressure
CAC: Coronary artery calcification
CARDIA: Coronary Artery Risk Development in Young Adults Study
cD: Carotid distensibility
CI: Confidence interval
CV: Cardiovascular
DBP: Diastolic blood pressure
ESH: European Society of Hypertension
FMD: Flow-mediated dilatation
GRS: Genetic risk score
HR: Hazard ratio
i3C: International Childhood Cardiovascular Cohort
IMT: Intima-media thickness
LV: Left ventricular
LVMI: Left ventricular mass index
NPV: Negative predicted value
OR: Odds ratio
PDAY: Pathobiological Determinants of Atherosclerosis in Youth
PPV: Positive predicted value
PRS: Polygenic risk score
PWV: Pulse wave velocity
SBP: Systolic blood pressure
SD: Standard deviation
SNP: Single-nucleotide-polymorphisms
UK: United Kingdom
US: United States
USPSTF: United States Preventive Services Task Force

## References

[1] McGill HC, McMahan CA, Gidding SS. Preventing heart disease in the 21st century: Implications of the Pathobiological Determinants of Atherosclerosis in Youth (PDAY) Study. Circulation. 2008;117(9):1216–27. 10.1161/CIRCULATIONAHA.107.717033. 18316498 10.1161/CIRCULATIONAHA.107.717033

[2] Berenson GS, Srinivasan SR, Bao W, Newman WP, Tracy RE, Wattigney WA. Association between multiple cardiovascular risk factors and atherosclerosis in children and young adults. The Bogalusa Heart Study. N Engl J Med. 1998;338(23):1650–6. 10.1056/NEJM199806043382302. 9614255 10.1056/NEJM199806043382302

[3] Napoli C, D’Armiento FP, Mancini FP, Postiglione A, Witztum JL, Palumbo G, et al. Fatty streak formation occurs in human fetal aortas and is greatly enhanced by maternal hypercholesterolemia. Intimal accumulation of low density lipoprotein and its oxidation precede monocyte recruitment into early atherosclerotic lesions. J Clin Invest. 1997;100(11):2680–90. 10.1172/jci119813. 9389731 10.1172/JCI119813 PMC508471

[4] Strong JP, McGill HC. The natural history of coronary atherosclerosis. Am J Pathol. 1962;40(1):37–49. 13917861 PMC1949572

[5] Chen X, Wang Y. Tracking of blood pressure from childhood to adulthood. Circulation. 2008;117(25):3171–80. 10.1161/circulationaha.107.730366. 18559702 10.1161/CIRCULATIONAHA.107.730366 PMC3568631

[6] Toschke AM, Kohl L, Mansmann U, von Kries R. Meta-analysis of blood pressure tracking from childhood to adulthood and implications for the design of intervention trials. Acta Paediatr. 2010;99(1):24–9. 10.1111/j.1651-2227.2009.01544.x. 19839954 10.1111/j.1651-2227.2009.01544.x

[7] Yang L, Magnussen CG, Yang L, Bovet P, Xi B. Elevated blood pressure in childhood or adolescence and cardiovascular outcomes in adulthood: A systematic review. Hypertension. 2020;75(4):948–55. 10.1161/HYPERTENSIONAHA.119.14168. **This systematic review and meta-analysis consolidate evidence from multiple studies to establish a clear link between elevated BP in youth and hypertension and target end-organ damage in adulthood, providing the basis for future study extending to overt clinical events**. 32114851 10.1161/HYPERTENSIONAHA.119.14168

[8] Magnussen CG, Smith KJ. Pediatric blood pressure and adult preclinical markers of cardiovascular disease. Clin Med Insights Blood Disord. 2016;9(4):1–8. 10.4137/cmbd.S18887. 27168729 10.4137/CMBD.S18887 PMC4857790

[9] Jacobs DR Jr, Woo JG, Sinaiko AR, Daniels SR, Ikonen J, Juonala M, et al. Childhood cardiovascular risk factors and adult cardiovascular events. N Engl J Med. 2022;386(20):1877–88. 10.1056/NEJMoa2109191. **This landmark study including 38,589 participants data from seven international cohorts provides compelling longitudinal evidence linking youth cardiovascular risk factors, including elevated BP, to adult cardiovascular events. This study reinforces the need for early intervention and management of cardiovascular risk factors, advocating for a proactive approach to cardiovascular health from a young age to prevent cardivasculr events in adulthood**. 35373933 10.1056/NEJMoa2109191 PMC9563825

[10] Fishman B, Bardugo A, Zloof Y, Bendor CD, Libruder C, Zucker I, et al. Adolescent hypertension is associated with stroke in young adulthood: A nationwide cohort of 1.9 million adolescents. Stroke. 2023;54(6):1531–7. 10.1161/STROKEAHA.122.042100. 37139816 10.1161/STROKEAHA.122.042100

[11] Sun J, Qiao Y, Zhao M, Magnussen CG, Xi B. Global, regional, and national burden of cardiovascular diseases in youths and young adults aged 15–39 years in 204 countries/territories, 1990–2019: A systematic analysis of Global Burden of Disease Study 2019. BMC Med. 2023;21(1):222. 10.1186/s12916-023-02925-4. 37365627 10.1186/s12916-023-02925-4 PMC10294522

[12] Daniels SR. On the US Preventive Services Task Force statement on screening for lipid disorders in children and adolescents: One step forward and 2 steps sideways. JAMA Pediatr. 2016;170(10):932–4. 10.1001/jamapediatrics.2016.2315. 27533098 10.1001/jamapediatrics.2016.2315

[13] Raitakari O, Pahkala K, Magnussen CG. Prevention of atherosclerosis from childhood. Nat Rev Cardiol. 2022;19(8):543–54. 10.1038/s41569-021-00647-9. **This review synthesizes a wide range of studies and data to provide a comprehensive overview of current understanding and recommendations for atherosclerosis prevention starting from early years**. 34987194 10.1038/s41569-021-00647-9

[14] de Ferranti SD, Moran AE, Kazi DS. Still “on the Fence” about universal childhood lipid screening: The USPSTF reaffirms an I statement. JAMA. 2023;330(3):225–7. 10.1001/jama.2023.11258. 37462716 10.1001/jama.2023.11258

[15] Chrysaidou K, Chainoglou A, Karava V, Dotis J, Printza N, Stabouli S. Secondary hypertension in children and adolescents: Novel insights. Curr Hypertens Rev. 2020;16(1):37–44. 10.2174/1573402115666190416152820. 31038068 10.2174/1573402115666190416152820

[16] Lurbe E, Redon J. Secondary Hypertension Clinical Presentation, Diagnosis, and Treatment. Mansoor GA, editor. 2004. p. 279–304.

[17] Flynn JT, Kaelber DC, Baker-Smith CM, Blowey D, Carroll AE, Daniels SR, et al. Clinical practice guideline for screening and management of high blood pressure in children and adolescents. Pediatrics. 2017;140(3):e20171904. 10.1542/peds.2017-1904. 28827377 10.1542/peds.2017-1904

[18] Lurbe E, Agabiti-Rosei E, Cruickshank JK, Dominiczak A, Erdine S, Hirth A, et al. 2016 European Society of Hypertension guidelines for the management of high blood pressure in children and adolescents. J Hypertens. 2016;34(10):1887–920. 10.1097/hjh.0000000000001039. 27467768 10.1097/HJH.0000000000001039

[19] National High Blood Pressure Education Program Working Group on High Blood Pressure in Children and Adolescents. The fourth report on the diagnosis, evaluation, and treatment of high blood pressure in children and adolescents. Pediatrics. 2004;114:555–76. 15286277

[20] Liu Q, Hou Y, Yang L, Zhao M, Li S, Xi B. Diagnostic effect of the single bp cut-offs for identifying elevated BP and hypertension in adolescents aged 13–17 years. Pediatr Cardiol. 2019;40(4):738–43. 10.1007/s00246-019-02058-7. 30707250 10.1007/s00246-019-02058-7

[21] de Simone G, Mancusi C, Hanssen H, Genovesi S, Lurbe E, Parati G, et al. Hypertension in children and adolescents. Eur Heart J. 2022;43(35):3290–301. 10.1093/eurheartj/ehac328. 35896123 10.1093/eurheartj/ehac328

[22] Writing Group of 2018 Chinese Guidelines for the Management of Hypertension. 2018 Chinese guidelines for the management of hypertension. Chin J Cardiol. 2019;24(1):24–56. 10.3969/j.issn.1007-5410.2019.01.002.

[23] Raj M, Sundaram R, Paul M, Kumar K. Blood pressure distribution in Indian children. Indian Pediatr. 2010;47(6):477–85. 10.1007/s13312-010-0089-z. 19736371 10.1007/s13312-010-0089-z

[24] Jackson LV, Thalange NK, Cole TJ. Blood pressure centiles for Great Britain. Arch Dis Child. 2007;92(4):298–303. 10.1136/adc.2005.081216. 16905566 10.1136/adc.2005.081216 PMC2083671

[25] Xi B, Zong X, Kelishadi R, Hong YM, Khadilkar A, Steffen LM, et al. Establishing international blood pressure references among nonoverweight children and adolescents aged 6 to 17 years. Circulation. 2016;133(4):398–408. 10.1161/circulationaha.115.017936. 26671979 10.1161/CIRCULATIONAHA.115.017936 PMC4729639

[26] Craig A, Breet Y, Gafane-Matemane LF, Norris SA, Kruger R. Detecting and managing childhood onset hypertension in Africa: A call to action. Curr Hypertens Rep. 2023. 10.1007/s11906-023-01247-3. **This review highlights the growing concern of hypertension among youth in Africa, an issue that has received limited attention despite its potential to significantly impact long-term health outcomes. The paper emphasizes the need for tailored detection and management strategies that consider the unique socioeconomic, cultural, and healthcare contexts of the low/middle-income areas**. 37318686 10.1007/s11906-023-01247-3 PMC10491553

[27] Rabi DM, McBrien KA, Sapir-Pichhadze R, Nakhla M, Ahmed SB, Dumanski SM, et al. Hypertension Canada’s 2020 comprehensive guidelines for the prevention, diagnosis, risk assessment, and treatment of hypertension in adults and children. Can J Cardiol. 2020;36(5):596–624. 10.1016/j.cjca.2020.02.086. 32389335 10.1016/j.cjca.2020.02.086

[28] Umemura S, Arima H, Arima S, Asayama K, Dohi Y, Hirooka Y, et al. The Japanese Society of Hypertension guidelines for the management of hypertension (JSH 2019). Hypertens Res. 2019;42(9):1235–481. 10.1038/s41440-019-0284-9. 31375757 10.1038/s41440-019-0284-9

[29] Xi B, Zhang T, Li S, Harville E, Bazzano L, He J, et al. Can pediatric hypertension criteria be simplified? A prediction analysis of subclinical cardiovascular outcomes from the Bogalusa Heart Study. Hypertension. 2017;69(4):691–6. 10.1161/hypertensionaha.116.08782. **This study found that a simplified childhood blood pressure definition associates with adult hypertension and subclinical cardiovascular disease that is equal to a complex definition does. The results of this study provide a basis for some blood pressure guidelines to propose a simplified definition of pediatric hypertension**. 28223474 10.1161/HYPERTENSIONAHA.116.08782 PMC5344730

[30] Yang L, Whincup PH, López-Bermejo A, Caserta CA, Muniz Medeiros CC, Kollias A, et al. Use of static cutoffs of hypertension to determine high cIMT in children and adolescents: An International Collaboration Study. Can J Cardiol. 2020;36(9):1467–73. 10.1016/j.cjca.2020.02.093. 32492399 10.1016/j.cjca.2020.02.093

[31] Zhao M, Mill JG, Yan WL, Hong YM, Skidmore P, Stoner L, et al. Static cut-points of hypertension and increased arterial stiffness in children and adolescents: The International Childhood Vascular Function Evaluation Consortium. J Clin Hypertens (Greenwich). 2019;21(9):1335–42. 10.1111/jch.13642. 31389662 10.1111/jch.13642 PMC8030373

[32] Ma C, Kelishadi R, Hong YM, Bovet P, Khadilkar A, Nawarycz T, et al. Performance of eleven simplified methods for the identification of elevated blood pressure in children and adolescents. Hypertension. 2016;68(3):614–20. 10.1161/hypertensionaha.116.07659. 27432869 10.1161/HYPERTENSIONAHA.116.07659 PMC4991624

[33] Hanevold CD, Brady TM. Screening and management of pediatric high blood pressure-challenges to implementing the clinical practice guideline. Curr Hypertens Rep. 2024;26(6):259–71. 10.1007/s11906-024-01298-0. Epub 2024 Mar 9. 38460067 10.1007/s11906-024-01298-0

[34] Stergiou GS, Boubouchairopoulou N, Kollias A. Accuracy of automated blood pressure measurement in children: Evidence, issues, and perspectives. Hypertension. 2017;69(6):1000–6. 10.1161/HYPERTENSIONAHA.116.08553. 28438903 10.1161/HYPERTENSIONAHA.116.08553

[35] Kollios K, Nika T, Kotsis V, Chrysaidou K, Antza C, Stabouli S. Arterial stiffness in children and adolescents with masked and sustained hypertension. J Hum Hypertens. 2021;35(1):85–93. 10.1038/s41371-020-0318-4. 32099080 10.1038/s41371-020-0318-4

[36] Lurbe E, Redon J, Alvarez J, Grau-Perez M, Martinez F, Mancia G. Insights from matched office and ambulatory blood pressure in youth: Clinical relevance. Hypertension. 2022;79(6):1237–46. 10.1161/HYPERTENSIONAHA.122.18993. 35345885 10.1161/HYPERTENSIONAHA.122.18993

[37] Swartz SJ, Srivaths PR, Croix B, Feig DI. Cost-effectiveness of ambulatory blood pressure monitoring in the initial evaluation of hypertension in children. Pediatrics. 2008;122(6):1177–81. 10.1542/peds.2007-3432. 19047231 10.1542/peds.2007-3432

[38] Sorof JM, Portman RJ. White coat hypertension in children with elevated casual blood pressure. J Pediatr. 2000;137(4):493–7. 10.1067/mpd.2000.108394. 11035827 10.1067/mpd.2000.108394

[39] Stabouli S, Kotsis V, Toumanidis S, Papamichael C, Constantopoulos A, Zakopoulos N. White-coat and masked hypertension in children: Association with target-organ damage. Pediatr Nephrol. 2005;20(8):1151–5. 10.1007/s00467-005-1979-5. 15947982 10.1007/s00467-005-1979-5

[40] Hamdani G, Flynn JT, Daniels S, Falkner B, Hanevold C, Ingelfinger J, et al. Ambulatory blood pressure monitoring tolerability and blood pressure status in adolescents: The SHIP AHOY study. Blood Press Monit. 2019;24(1):12–7. 10.1097/mbp.0000000000000354. 30451702 10.1097/MBP.0000000000000354 PMC6398596

[41] Flynn JT, Urbina EM, Brady TM, Baker-Smith C, Daniels SR, Hayman LL, et al. Ambulatory blood pressure monitoring in children and adolescents: 2022 Update: A scientific statement from the American Heart Association. Hypertension. 2022;79(7):e114–24. 10.1161/HYP.0000000000000215. 35603599 10.1161/HYP.0000000000000215 PMC12168719

[42] Lurbe E, Torro I, Alvarez V, Nawrot T, Paya R, Redon J, et al. Prevalence, persistence, and clinical significance of masked hypertension in youth. Hypertension. 2005;45(4):493–8. 10.1161/01.HYP.0000160320.39303.ab. 15767467 10.1161/01.HYP.0000160320.39303.ab

[43] Chung J, Robinson C, Sheffield L, Paramanathan P, Yu A, Ewusie J, et al. Prevalence of pediatric masked hypertension and risk of subclinical cardiovascular outcomes: a systematic review and meta-analysis. Hypertension. 2023;80(11):2280–92. 10.1161/HYPERTENSIONAHA.123.20967. 37737026 10.1161/HYPERTENSIONAHA.123.20967

[44] Stergiou G, Stambolliu E, Bountzona I, Ntineri A, Kollias A, Vazeou A, et al. Home blood pressure monitoring in children and adolescents: Systematic review of evidence on clinical utility. Curr Hypertens Rep. 2019;21(8):64. 10.1007/s11906-019-0967-2. 31240404 10.1007/s11906-019-0967-2

[45] Song P, Zhang Y, Yu J, Zha M, Zhu Y, Rahimi K, et al. Global prevalence of hypertension in children: A systematic review and meta-analysis. JAMA Pediatr. 2019;173(12):1154–63. 10.1001/jamapediatrics.2019.3310. 31589252 10.1001/jamapediatrics.2019.3310 PMC6784751

[46] Hardy ST, Sakhuja S, Jaeger BC, Urbina EM, Suglia SF, Feig DI, et al. Trends in blood pressure and hypertension among US children and adolescents, 1999–2018. JAMA Netw Open. 2021;4(4):e213917. 10.1001/jamanetworkopen.2021.3917. 33792732 10.1001/jamanetworkopen.2021.3917 PMC8017470

[47] Wang L, Song L, Liu B, Zhang L, Wu M, Cao Z, et al. Trends and status of the prevalence of elevated blood pressure in children and adolescents in China: A systematic review and meta-analysis. Curr Hypertens Rep. 2019;21(11):88. 10.1007/s11906-019-0992-1. 31599364 10.1007/s11906-019-0992-1

[48] Park PG, Park E, Kang HG. Increasing trend in hypertension prevalence among Korean adolescents from 2007 to 2020. BMC Public Health. 2024;24(1):617. 10.1186/s12889-024-18093-w. 38409007 10.1186/s12889-024-18093-w PMC10898016

[49] Meena J, Singh M, Agarwal A, Chauhan A, Jaiswal N. Prevalence of hypertension among children and adolescents in India: A systematic review and meta-analysis. Indian J Pediatr. 2021;88(11):1107–14. 10.1007/s12098-021-03686-9. 33796994 10.1007/s12098-021-03686-9

[50] Noubiap JJ, Essouma M, Bigna JJ, Jingi AM, Aminde LN, Nansseu JR. Prevalence of elevated blood pressure in children and adolescents in Africa: A systematic review and meta-analysis. Lancet Public Health. 2017;2(8):e375–86. 10.1016/s2468-2667(17)30123-8. 29253478 10.1016/S2468-2667(17)30123-8

[51] Crouch SH, Soepnel LM, Kolkenbeck-Ruh A, Maposa I, Naidoo S, Davies J, et al. Paediatric hypertension in Africa: A Systematic Review and Meta-Analysis. EClinicalMedicine. 2022;43:101229. 10.1016/j.eclinm.2021.101229. 34917909 10.1016/j.eclinm.2021.101229 PMC8665406

[52] Xi B, Bovet P, Hong YM, Zong X, Chiolero A, Kim HS, et al. Recent blood pressure trends in adolescents from China, Korea, Seychelles and the United States of America, 1997–2012. J Hypertens. 2016;34(10):1948–58. 10.1097/hjh.0000000000001058. 27467766 10.1097/HJH.0000000000001058

[53] DelRosso LM, Mogavero MP, Ferri R. Effect of sleep disorders on blood pressure and hypertension in children. Curr Hypertens Rep. 2020;22(11):88. 10.1007/s11906-020-01100-x. 32893326 10.1007/s11906-020-01100-x

[54] Sparano S, Lauria F, Ahrens W, Fraterman A, Thumann B, Iacoviello L, et al. Sleep duration and blood pressure in children: Analysis of the pan-European IDEFICS cohort. J Clin Hypertens (Greenwich). 2019;21(5):572–8. 10.1111/jch.13520. 30892825 10.1111/jch.13520 PMC8030491

[55] Ai S, Li Z, Wang S, Chen S, Chan JW, Au CT, et al. Blood pressure and childhood obstructive sleep apnea: A systematic review and meta-analysis. Sleep Med Rev. 2022;65:101663. 10.1016/j.smrv.2022.101663. 36087456 10.1016/j.smrv.2022.101663

[56] Whiting S, Buoncristiano M, Gelius P, Abu-Omar K, Pattison M, Hyska J, et al. Physical activity, screen time, and sleep duration of children aged 6–9 years in 25 countries: An analysis within the WHO European Childhood Obesity Surveillance Initiative (COSI) 2015–2017. Obes Facts. 2021;14(1):32–44. 10.1159/000511263. 33352575 10.1159/000511263 PMC7983588

[57] Levy RV, Brathwaite KE, Sarathy H, Reidy K, Kaskel FJ, Melamed ML. Analysis of active and passive tobacco exposures and blood pressure in US children and adolescents. JAMA Netw Open. 2021;4(2):e2037936. 10.1001/jamanetworkopen.2020.37936. 33620445 10.1001/jamanetworkopen.2020.37936 PMC7903259

[58] Huang M, Chen J, Yang Y, Yuan H, Huang Z, Lu Y. Effects of ambient air pollution on blood pressure among children and adolescents: A systematic review and meta-analysis. J Am Heart Assoc. 2021;10(10):e017734. 10.1161/jaha.120.017734. 33942625 10.1161/JAHA.120.017734 PMC8200690

[59] Shen W, Zhang T, Li S, Zhang H, Xi B, Shen H, et al. Race and sex differences of long-term blood pressure profiles from childhood and adult hypertension: The Bogalusa Heart Study. Hypertension. 2017;70(1):66–74. 10.1161/hypertensionaha.117.09537. 28533330 10.1161/HYPERTENSIONAHA.117.09537 PMC5711390

[60] Wang X, Xu X, Su S, Snieder H. Familial aggregation and childhood blood pressure. Curr Hypertens Rep. 2015;17(1):509. 10.1007/s11906-014-0509-x. 25432901 10.1007/s11906-014-0509-x PMC4476022

[61] Gu C, Borecki I, Gagnon J, Bouchard C, Leon AS, Skinner JS, et al. Familial resemblance for resting blood pressure with particular reference to racial differences: Preliminary analyses from the HERITAGE Family Study. Hum Biol. 1998;70(1):77–90. 9489236

[62] Wang X, Xu X, Su S, Snieder H. Familial aggregation of blood pressure. Curr Hypertens Rep. 2015;17(1):195–209. 10.1007/s11906-014-0509-x. PMID: 25432901. 10.1007/s11906-014-0509-x PMC4476022 25432901

[63] Niiranen TJ, McCabe EL, Larson MG, Henglin M, Lakdawala NK, Vasan RS, et al. Heritability and risks associated with early onset hypertension: multigenerational, prospective analysis in the Framingham Heart Study. BMJ. 2017;357:j1949. 10.1136/bmj.j1949. **The study used extensive multigenerational data from the Framingham Heart Study, offering a unique insights into how genetic and environmental factors contribute to the early onset of hypertension, distinguishing it from later-onset forms of the condition. The findings underscore the significant genetic hereditary risk associated with early onset hypertension, highlighting the importance of family history in assessing individual risk profiles. This has profound implications for clinical practice, emphasizing the need for early screening and intervention strategies in individuals with a family history of early onset hypertension**. 28500036 10.1136/bmj.j1949 PMC5430541

[64] Niinikoski H, Jula A, Viikari J, Rönnemaa T, Heino P, Lagström H, et al. Blood pressure is lower in children and adolescents with a low-saturated-fat diet since infancy: The Special Turku Coronary Risk Factor Intervention Project. Hypertension. 2009;53(6):918–24. 10.1161/hypertensionaha.109.130146. 19364991 10.1161/HYPERTENSIONAHA.109.130146

[65] Burke V, Gracey MP, Beilin LJ, Milligan RA. Family history as a predictor of blood pressure in a longitudinal study of Australian children. J Hypertens. 1998;16(3):269–76. 10.1097/00004872-199816030-00003. 9557919 10.1097/00004872-199816030-00003

[66] Juhola J, Oikonen M, Magnussen CG, Mikkilä V, Siitonen N, Jokinen E, et al. Childhood physical, environmental, and genetic predictors of adult hypertension: The cardiovascular risk in young Finns study. Circulation. 2012;126(4):402–9. 10.1161/circulationaha.111.085977. 22718800 10.1161/CIRCULATIONAHA.111.085977

[67] Cheng CF, Hsieh AR, Liang WM, Chen CC, Chen CH, Wu JY, et al. Genome-wide and candidate gene association analyses identify a 14-SNP combination for hypertension in patients with type 2 diabetes. Am J Hypertens. 2021;34(6):651–61. 10.1093/ajh/hpaa203. 33276381 10.1093/ajh/hpaa203

[68] Evangelou E, Warren HR, Mosen-Ansorena D, Mifsud B, Pazoki R, Gao H, et al. Genetic analysis of over 1 million people identifies 535 new loci associated with blood pressure traits. Nat Genet. 2018;50(10):1412–25. 10.1038/s41588-018-0205-x. 30224653 10.1038/s41588-018-0205-x PMC6284793

[69] Oikonen M, Tikkanen E, Juhola J, Tuovinen T, Seppälä I, Juonala M, et al. Genetic variants and blood pressure in a population-based cohort: The Cardiovascular Risk in Young Finns study. Hypertension. 2011;58(6):1079–85. 10.1161/hypertensionaha.111.179291. 22025373 10.1161/HYPERTENSIONAHA.111.179291 PMC3247907

[70] Howe LD, Parmar PG, Paternoster L, Warrington NM, Kemp JP, Briollais L, et al. Genetic influences on trajectories of systolic blood pressure across childhood and adolescence. Circ Cardiovasc Genet. 2013;6(6):608–14. 10.1161/circgenetics.113.000197. 24200906 10.1161/CIRCGENETICS.113.000197 PMC4759925

[71] Parmar PG, Taal HR, Timpson NJ, Thiering E, Lehtimäki T, Marinelli M, et al. International genome-wide association study consortium identifies novel loci associated with blood pressure in children and adolescents. Circ Cardiovasc Genet. 2016;9(3):266–78. 10.1161/circgenetics.115.001190. 26969751 10.1161/CIRCGENETICS.115.001190 PMC5279885

[72] Pérez-Gimeno G, Seral-Cortes M, Sabroso-Lasa S, Esteban LM, Lurbe E, Béghin L, et al. Development of a genetic risk score to predict the risk of hypertension in European adolescents from the HELENA study. Front Cardiovasc Med. 2023;10:1118919. 10.3389/fcvm.2023.1118919. 37324619 10.3389/fcvm.2023.1118919 PMC10267871

[73] Yang L, Sun J, Zhao M, Liang Y, Bovet P, Xi B. Elevated blood pressure in childhood and hypertension risk in adulthood: A systematic review and meta-analysis. J Hypertens. 2020;38(12):2346–55. 10.1097/hjh.0000000000002550. 32649636 10.1097/HJH.0000000000002550

[74] Gartlehner G, Vander Schaaf EB, Orr C, Kennedy SM, Clark R, Viswanathan M. Screening for hypertension in children and adolescents: Updated evidence report and systematic review for the US Preventive Services Task Force. JAMA. 2020;324(18):1884–95. 10.1001/jama.2020.11119. **This review provided an extensive synthesis of the available evidence on screening practices for hypertension in children and adolescents. This study also identifies gaps in the current research landscape, setting the stage for future studies and interventions aimed at optimizing hypertension screening and management in children and adolescents**. 33170247 10.1001/jama.2020.11119

[75] Du T, Fernandez C, Barshop R, Chen W, Urbina EM, Bazzano LA. 2017 pediatric hypertension guidelines improve prediction of adult cardiovascular outcomes. Hypertension. 2019;73(6):1217–23. 10.1161/hypertensionaha.118.12469. 31006329 10.1161/HYPERTENSIONAHA.118.12469 PMC6673663

[76] Wills AK, Lawlor DA, Matthews FE, Sayer AA, Bakra E, Ben-Shlomo Y, et al. Life course trajectories of systolic blood pressure using longitudinal data from eight UK cohorts. PLoS Med. 2011;8(6):e1000440. 10.1371/journal.pmed.1000440. 21695075 10.1371/journal.pmed.1000440 PMC3114857

[77] Twisk J, Hoekstra T. Classifying developmental trajectories over time should be done with great caution: a comparison between methods. J Clin Epidemiol. 2012;65(10):1078–87. 10.1016/j.jclinepi.2012.04.010. 22818946 10.1016/j.jclinepi.2012.04.010

[78] Haley JE, Woodly SA, Daniels SR, Falkner B, Ferguson MA, Flynn JT, et al. Association of blood pressure-related increase in vascular stiffness on other measures of target organ damage in youth. Hypertension. 2022;79(9):2042–50. 10.1161/hypertensionaha.121.18765. 35762327 10.1161/HYPERTENSIONAHA.121.18765 PMC9378473

[79] Li S, Chen W, Srinivasan SR, Berenson GS. Childhood blood pressure as a predictor of arterial stiffness in young adults: The Bogalusa Heart Study. Hypertension. 2004;43(3):541–6. 10.1161/01.HYP.0000115922.98155.23. 14744922 10.1161/01.HYP.0000115922.98155.23

[80] Juonala M, Järvisalo MJ, Mäki-Torkko N, Kähönen M, Viikari JSA, Raitakari OT. Risk factors identified in childhood and decreased carotid artery elasticity in adulthood: The Cardiovascular Risk in Young Finns Study. Circulation. 2005;112(10):1486–93. 10.1161/circulationaha.104.502161. 16129802 10.1161/CIRCULATIONAHA.104.502161

[81] Aatola H, Hutri-Kähönen N, Juonala M, Viikari JSA, Hulkkonen J, Laitinen T, et al. Lifetime risk factors and arterial pulse wave velocity in adulthood: The Cardiovascular Risk in Young Finns Study. Hypertension. 2010;55(3):806–11. 10.1161/hypertensionaha.109.145102. 20083727 10.1161/HYPERTENSIONAHA.109.145102

[82] Aatola H, Magnussen CG, Koivistoinen T, Hutri-Kähönen N, Juonala M, Viikari JS, et al. Simplified definitions of elevated pediatric blood pressure and high adult arterial stiffness. Pediatrics. 2013;132(1):e70–6. 10.1542/peds.2012-3426. 23753088 10.1542/peds.2012-3426

[83] Li S, Chen W, Yun M, Fernandez C, Krousel-Wood M, Webber L, et al. Sex and race (Black-White) differences in the relationship of childhood risk factors to adulthood arterial stiffness: The Bogalusa Heart Study. Am J Med Sci. 2014;348(2):101–7. 10.1097/maj.0000000000000264. 24762753 10.1097/MAJ.0000000000000264 PMC4108517

[84] Liang Y, Hou D, Shan X, Zhao X, Hu Y, Jiang B, et al. Cardiovascular remodeling relates to elevated childhood blood pressure: Beijing Blood Pressure Cohort Study. Int J Cardiol. 2014;177(3):836–9. 10.1016/j.ijcard.2014.11.013. 25465829 10.1016/j.ijcard.2014.11.013

[85] Chu C, Dai Y, Mu J, Yang R, Wang M, Yang J, et al. Associations of risk factors in childhood with arterial stiffness 26 years later: The Hanzhong Adolescent Hypertension Cohort. J Hypertens. 2017;35(Suppl 1):S10–5. 10.1097/hjh.0000000000001242. 28060189 10.1097/HJH.0000000000001242

[86] Juonala M, Magnussen CG, Venn A, Dwyer T, Burns TL, Davis PH, et al. Influence of age on associations between childhood risk factors and carotid intima-media thickness in adulthood. Circulation. 2010;122(24):2514–20. 10.1161/circulationaha.110.966465. 21126976 10.1161/CIRCULATIONAHA.110.966465

[87] Koskinen J, Juonala M, Dwyer T, Venn A, Thomson R, Bazzano L, et al. Impact of lipid measurements in youth in addition to conventional clinic-based risk factors on predicting preclinical atherosclerosis in adulthood: International Childhood Cardiovascular Cohort Consortium. Circulation. 2018;137(12):1246–55. 10.1161/circulationaha.117.029726. 29170152 10.1161/CIRCULATIONAHA.117.029726 PMC5860965

[88] Koskinen J, Juonala M, Dwyer T, Venn A, Petkeviciene J, Čeponienė I, et al. Utility of different blood pressure measurement components in childhood to predict adult carotid intima-media thickness. Hypertension. 2019;73(2):335–41. 10.1161/hypertensionaha.118.12225. 30580683 10.1161/HYPERTENSIONAHA.118.12225 PMC6326843

[89] Yan Y, Hou D, Liu J, Zhao X, Cheng H, Xi B, et al. Childhood body mass index and blood pressure in prediction of subclinical vascular damage in adulthood: Beijing Blood Pressure Cohort. J Hypertens. 2017;35(1):47–54. 10.1097/hjh.0000000000001118. 27648721 10.1097/HJH.0000000000001118

[90] Davis PH, Dawson JD, Riley WA, Lauer RM. Carotid intimal-medial thickness is related to cardiovascular risk factors measured from childhood through middle age. The Muscatine Study Circulation. 2001;104(23):2815–9. 10.1161/hc4601.099486. 11733400 10.1161/hc4601.099486

[91] Li S, Chen W, Srinivasan SR, Tang R, Bond MG, Berenson GS. Race (black-white) and gender divergences in the relationship of childhood cardiovascular risk factors to carotid artery intima-media thickness in adulthood: The Bogalusa Heart Study. Atherosclerosis. 2007;194(2):421–5. 10.1016/j.atherosclerosis.2006.08.026. 17123535 10.1016/j.atherosclerosis.2006.08.026

[92] Ceponiene I, Klumbiene J, Tamuleviciute-Prasciene E, Motiejunaite J, Sakyte E, Ceponis J, et al. Associations between risk factors in childhood (12–13 years) and adulthood (48–49 years) and subclinical atherosclerosis: The Kaunas Cardiovascular Risk Cohort Study. BMC Cardiovasc Disord. 2015;15:89. 10.1186/s12872-015-0087-0. 26282122 10.1186/s12872-015-0087-0 PMC4539717

[93] Dawson JD, Sonka M, Blecha MB, Lin W, Davis PH. Risk factors associated with aortic and carotid intima-media thickness in adolescents and young adults. J Am Coll Cardiol. 2009;53(24):2273–9. 10.1016/j.jacc.2009.03.026. 19520251 10.1016/j.jacc.2009.03.026 PMC2747309

[94] Mahoney LT, Burns TL, Stanford W, Thompson BH, Witt JD, Rost CA, et al. Coronary risk factors measured in childhood and young adult life are associated with coronary artery calcification in young adults: The Muscatine Study. J Am Coll Cardiol. 1996;27(2):277–84. 10.1016/0735-1097(95)00461-0. 8557894 10.1016/0735-1097(95)00461-0

[95] Hartiala O, Magnussen CG, Kajander S, Knuuti J, Ukkonen H, Saraste A, et al. Adolescence risk factors are predictive of coronary artery calcification at middle age: The Cardiovascular Risk in Young Finns Study. J Am Coll Cardiol. 2012;60(15):1364–70. 10.1016/j.jacc.2012.05.045. 22981553 10.1016/j.jacc.2012.05.045

[96] Juonala M, Viikari JS, Rönnemaa T, Helenius H, Taittonen L, Raitakari OT. Elevated blood pressure in adolescent boys predicts endothelial dysfunction: The Cardiovascular Risk in Young Finns Study. Hypertension. 2006;48(3):424–30. 10.1161/01.Hyp.0000237666.78217.47. 16880344 10.1161/01.HYP.0000237666.78217.47

[97] Sinha MD, Azukaitis K, Sladowska-Kozłowska J, Bårdsen T, Merkevicius K, Karlsen Sletten IS, et al. Prevalence of left ventricular hypertrophy in children and young people with primary hypertension: Meta-analysis and meta-regression. Front Cardiovasc Med. 2022;9: 993513. 10.3389/fcvm.2022.993513. 36386367 10.3389/fcvm.2022.993513 PMC9659762

[98] Toprak A, Wang H, Chen W, Paul T, Srinivasan S, Berenson G. Relation of childhood risk factors to left ventricular hypertrophy (eccentric or concentric) in relatively young adulthood (from the Bogalusa Heart Study). Am J Cardiol. 2008;101(11):1621–5. 10.1016/j.amjcard.2008.01.045. 18489940 10.1016/j.amjcard.2008.01.045

[99] Magnussen CG, Dwyer T, Venn A. Family history of premature coronary heart disease, child cardio-metabolic risk factors and left ventricular mass. Cardiol Young. 2014;24(5):938–40. 10.1017/s1047951113001571. 24107484 10.1017/S1047951113001571

[100] Leiba A, Twig G, Vivante A, Skorecki K, Golan E, Derazne E, et al. Prehypertension among 2.19 million adolescents and future risk for end-stage renal disease. J Hypertens. 2017;35(6):1290–6. 10.1097/hjh.0000000000001295. 28169886 10.1097/HJH.0000000000001295

[101] Rovio SP, Pahkala K, Nevalainen J, Juonala M, Salo P, Kähönen M, et al. Cardiovascular risk factors from childhood and midlife cognitive performance: The Young Finns Study. J Am Coll Cardiol. 2017;69(18):2279–89. 10.1016/j.jacc.2017.02.060. 28473132 10.1016/j.jacc.2017.02.060

[102] Hakala JO, Pahkala K, Juonala M, Salo P, Kähönen M, Hutri-Kähönen N, et al. Cardiovascular risk factor trajectories since childhood and cognitive performance in midlife: The Cardiovascular Risk in Young Finns Study. Circulation. 2021;143(20):1949–61. 10.1161/circulationaha.120.052358. 33966448 10.1161/CIRCULATIONAHA.120.052358

[103] Flynn JT. What level of blood pressure is concerning in childhood? Circ Res. 2022;130(5):800–8. 10.1161/circresaha.121.319819. 35239405 10.1161/CIRCRESAHA.121.319819

[104] Juhola J, Magnussen CG, Berenson GS, Venn A, Burns TL, Sabin MA, et al. Combined effects of child and adult elevated blood pressure on subclinical atherosclerosis: The International Childhood Cardiovascular Cohort Consortium. Circulation. 2013;128(3):217–24. 10.1161/circulationaha.113.001614. **This study, which included 4,210 participants from four international cohorts, demonstrated that individuals with persistently elevated blood pressure from childhood to adulthood are at an increased risk of carotid atherosclerosis. However, if high blood pressure during childhood resolves by adulthood, this risk is significantly reduced. In the absence of long-term clinical trials, this research underscores the potential benefits of achieving and maintaining normal blood pressure from an early age**. 23780579 10.1161/CIRCULATIONAHA.113.001614 PMC3875837

[105] Aatola H, Koivistoinen T, Tuominen H, Juonala M, Lehtimäki T, Viikari JSA, et al. Influence of child and adult elevated blood pressure on adult arterial stiffness: The Cardiovascular Risk in Young Finns Study. Hypertension. 2017;70(3):531–6. 10.1161/hypertensionaha.117.09444. 28674036 10.1161/HYPERTENSIONAHA.117.09444

[106] Kelly RK, Thomson R, Smith KJ, Dwyer T, Venn A, Magnussen CG. Factors affecting tracking of blood pressure from childhood to adulthood: The Childhood Determinants of Adult Health Study. J Pediatr. 2015;167(6):1422–8.e2. 10.1016/j.jpeds.2015.07.055. 26342719 10.1016/j.jpeds.2015.07.055

[107] Fan B, Zhang T, Li S, Yan Y, Fan L, Bazzano L, et al. Differential roles of life-course cumulative burden of cardiovascular risk factors in arterial stiffness and thickness. Can J Cardiol. 2022;38(8):1253–62. 10.1016/j.cjca.2022.03.009. 35314334 10.1016/j.cjca.2022.03.009

[108] Li X, Li S, Ulusoy E, Chen W, Srinivasan SR, Berenson GS. Childhood adiposity as a predictor of cardiac mass in adulthood. Circulation. 2004;110(22):3488–92. 10.1161/01.cir.0000149713.48317.27. 15557363 10.1161/01.CIR.0000149713.48317.27

[109] Lai CC, Sun D, Cen R, Wang J, Li S, Fernandez-Alonso C, et al. Impact of long-term burden of excessive adiposity and elevated blood pressure from childhood on adulthood left ventricular remodeling patterns: The Bogalusa Heart Study. J Am Coll Cardiol. 2014;64(15):1580–7. 10.1016/j.jacc.2014.05.072. 25301461 10.1016/j.jacc.2014.05.072 PMC4194028

[110] Heiskanen JS, Hernesniemi JA, Ruohonen S, Hutri-Kähönen N, Kähönen M, Jokinen E, et al. Influence of early-life body mass index and systolic blood pressure on left ventricle in adulthood – The Cardiovascular Risk in Young Finns Study. Ann Med. 2021;53(1):160–8. 10.1080/07853890.2020.1849785. 33238748 10.1080/07853890.2020.1849785 PMC7877918

[111] Hao G, Wang X, Treiber FA, Harshfield G, Kapuku G, Su S. Blood pressure trajectories from childhood to young adulthood associated with cardiovascular risk: Results from the 23-year longitudinal Georgia Stress and Heart Study. Hypertension. 2017;69(3):435–42. 10.1161/hypertensionaha.116.08312. 28093467 10.1161/HYPERTENSIONAHA.116.08312 PMC5300982

[112] Allen NB, Siddique J, Wilkins JT, Shay C, Lewis CE, Goff DC, et al. Blood pressure trajectories in early adulthood and subclinical atherosclerosis in middle age. JAMA. 2014;311(5):490–7. 10.1001/jama.2013.285122. 24496536 10.1001/jama.2013.285122 PMC4122296

[113] Meng Y, Sharman JE, Koskinen JS, Juonala M, Viikari JSA, Buscot MJ, et al. Blood pressure at different life stages over the early life course and intima-media thickness. JAMA Pediatr. 2024;178(2):133–41. 10.1001/jamapediatrics.2023.5351. **This study showed that the relative contribution of blood pressure to intima-media thickness is similar across the life-stages observed, which included infancy and preschool childhood. The findings reinforced the need for blood pressure prevention strategies in early life**. 38048127 10.1001/jamapediatrics.2023.5351 PMC10696511

[114] Meng Y, Buscot MJ, Juonala M, Wu F, Armstrong MK, Fraser BJ, et al. Relative contribution of blood pressure in childhood, young- and mid-adulthood to large artery stiffness in mid-adulthood. J Am Heart Assoc. 2022;11(12):e024394. 10.1161/jaha.121.024394. 35699171 10.1161/JAHA.121.024394 PMC9238667

[115] Suvila K, McCabe EL, Lehtonen A, Ebinger JE, Lima JAC, Cheng S, et al. Early onset hypertension is associated with hypertensive end-organ damage already by midlife. Hypertension. 2019;74(2):305–12. 10.1161/hypertensionaha.119.13069. 31256722 10.1161/HYPERTENSIONAHA.119.13069 PMC6938569

[116] Falkstedt D, Koupil I, Hemmingsson T. Blood pressure in late adolescence and early incidence of coronary heart disease and stroke in the Swedish 1969 conscription cohort. J Hypertens. 2008;26(7):1313–20. 10.1097/HJH.0b013e3282ffb17e. 18551005 10.1097/HJH.0b013e3282ffb17e

[117] Högström G, Nordström A, Eriksson M, Nordström P. Risk factors assessed in adolescence and the later risk of stroke in men: A 33-year follow-up study. Cerebrovasc Dis. 2015;39(1):63–71. 10.1159/000369960. 25547343 10.1159/000369960

[118] Morrison JA, Glueck CJ, Wang P. Childhood risk factors predict cardiovascular disease, impaired fasting glucose plus type 2 diabetes mellitus, and high blood pressure 26 years later at a mean age of 38 years: The Princeton-Lipid Research Clinics Follow-Up Study. Metabolism. 2012;61(4):531–41. 10.1016/j.metabol.2011.08.010. 22001337 10.1016/j.metabol.2011.08.010 PMC3324938

[119] Paffenbarger RS Jr, Wing AL. Characteristics in youth predisposing to fatal stroke in later years. Lancet. 1967;1(7493):753–4. 10.1016/s0140-6736(67)91367-0. 4164123 10.1016/s0140-6736(67)91367-0

[120] Gray L, Lee IM, Sesso HD, Batty GD. Blood pressure in early adulthood, hypertension in middle age, and future cardiovascular disease mortality: HAHS (Harvard Alumni Health Study). J Am Coll Cardiol. 2011;58(23):2396–403. 10.1016/j.jacc.2011.07.045. 22115646 10.1016/j.jacc.2011.07.045 PMC3253414

[121] Leiba A, Twig G, Levine H, Goldberger N, Afek A, Shamiss A, et al. Hypertension in late adolescence and cardiovascular mortality in midlife: A cohort study of 2.3 million 16- to 19-year-old examinees. Pediatr Nephrol. 2016;31(3):485–92. 10.1007/s00467-015-3240-1. 26508439 10.1007/s00467-015-3240-1

[122] Sundström J, Neovius M, Tynelius P, Rasmussen F. Association of blood pressure in late adolescence with subsequent mortality: Cohort study of Swedish male conscripts. BMJ. 2011;342:d643. 10.1136/bmj.d643. 21343202 10.1136/bmj.d643 PMC3042737

[123] Franks PW, Hanson RL, Knowler WC, Sievers ML, Bennett PH, Looker HC. Childhood obesity, other cardiovascular risk factors, and premature death. N Engl J Med. 2010;362(6):485–93. 10.1056/NEJMoa0904130. 20147714 10.1056/NEJMoa0904130 PMC2958822

[124] Robinson CH, Hussain J, Jeyakumar N, Smith G, Birken CS, Dart A, et al. Long-term cardiovascular outcomes in children and adolescents with hypertension. JAMA Pediatr. 2024. 10.1001/jamapediatrics.2024.1543. 38709137 10.1001/jamapediatrics.2024.1543 PMC11217870

[125] Nwabuo CC, Appiah D, Moreira HT, Vasconcellos HD, Yano Y, Reis JP, et al. Long-term cumulative blood pressure in young adults and incident heart failure, coronary heart disease, stroke, and cardiovascular disease: The CARDIA Study. Eur J Prev Cardiol. 2021;28(13):1445–51. 10.1177/2047487320915342. 34695218 10.1177/2047487320915342 PMC8653578

[126] Ference BA, Bhatt DL, Catapano AL, Packard CJ, Graham I, Kaptoge S, et al. Association of genetic variants related to combined exposure to lower low-density lipoproteins and lower systolic blood pressure with lifetime risk of cardiovascular disease. JAMA. 2019;322(14):1381–91. 10.1001/jama.2019.14120. 31475726 10.1001/jama.2019.14120 PMC6724415

[127] Foti K, Wang D, Appel LJ, Selvin E. Hypertension Awareness, treatment, and control in US adults: Trends in the hypertension control cascade by population subgroup (National Health and Nutrition Examination Survey, 1999–2016). Am J Epidemiol. 2019;188(12):2165–74. 10.1093/aje/kwz177. 31504121 10.1093/aje/kwz177 PMC7212399

[128] He FJ, MacGregor GA. Importance of salt in determining blood pressure in children: Meta-analysis of controlled trials. Hypertension. 2006;48(5):861–9. 10.1161/01.HYP.0000245672.27270.4a. 17000923 10.1161/01.HYP.0000245672.27270.4a

[129] Reed KE, Warburton DE, Macdonald HM, Naylor PJ, McKay HA. Action Schools! BC: A school-based physical activity intervention designed to decrease cardiovascular disease risk factors in children. Prev Med. 2008;46(6):525–31. 10.1016/j.ypmed.2008.02.020. 18377970 10.1016/j.ypmed.2008.02.020

[130] Eddolls WTB, McNarry MA, Stratton G, Winn CON, Mackintosh KA. High-intensity interval training interventions in children and adolescents: A systematic review. Sports Med. 2017;47(11):2363–74. 10.1007/s40279-017-0753-8. 28643209 10.1007/s40279-017-0753-8 PMC5633633

[131] Cai L, Wu Y, Wilson RF, Segal JB, Kim MT, Wang Y. Effect of childhood obesity prevention programs on blood pressure: A systematic review and meta-analysis. Circulation. 2014;129(18):1832–9. 10.1161/circulationaha.113.005666. 24552832 10.1161/CIRCULATIONAHA.113.005666 PMC4346224

[132] Alzahrani HS, Jackson KG, Hobbs DA, Lovegrove JA. The role of dietary nitrate and the oral microbiome on blood pressure and vascular tone. Nutr Res Rev. 2021;34(2):222–39. 10.1017/s0954422420000281. 33280615 10.1017/S0954422420000281

[133] Schaare HL, Blöchl M, Kumral D, Uhlig M, Lemcke L, Valk SL, et al. Associations between mental health, blood pressure and the development of hypertension. Nat Commun. 2023;14(1):1953. 10.1038/s41467-023-37579-6. 37029103 10.1038/s41467-023-37579-6 PMC10082210

[134] Rosenberger ME, Fulton JE, Buman MP, Troiano RP, Grandner MA, Buchner DM, et al. The 24-hour activity cycle: A new paradigm for physical activity. Med Sci Sports Exerc. 2019;51(3):454–64. 10.1249/mss.0000000000001811. 30339658 10.1249/MSS.0000000000001811 PMC6377291

[135] Inam M, Samad Z, Vaughan EM, Almas A, Hanif B, Minhas AM, et al. Global cardiovascular research: Gaps and opportunities. Curr Cardiol Rep. 2023;25(12):1831–8. 10.1007/s11886-023-01996-2. 37982934 10.1007/s11886-023-01996-2 PMC12413471

[136] Burrello J, Erhardt EM, Saint-Hilary G, Veglio F, Rabbia F, Mulatero P, et al. Pharmacological treatment of arterial hypertension in children and adolescents: A network meta-analysis. Hypertension. 2018;72(2):306–13. 10.1161/hypertensionaha.118.10862. 29967035 10.1161/HYPERTENSIONAHA.118.10862

[137] Samuel JP, Tyson JE, Green C, Bell CS, Pedroza C, Molony D, et al. Treating hypertension in children with n-of-1 trials. Pediatrics. 2019;143(4): e20181818. 10.1542/peds.2018-1818. 30842257 10.1542/peds.2018-1818 PMC6564074

[138] Kupferman JC, Paterno K, Mahgerefteh J, Pagala M, Golden M, Lytrivi ID, et al. Improvement of left ventricular mass with antihypertensive therapy in children with hypertension. Pediatr Nephrol. 2010;25(8):1513–8. 10.1007/s00467-010-1511-4. 20393750 10.1007/s00467-010-1511-4

[139] U.S. Preventive Services Task Force. Screening for high blood pressure: Recommendations and rationale. Am J Prev Med. 2003;25(2):159–64. 10.1016/s0749-3797(03)00122-3. 12880885 10.1016/s0749-3797(03)00122-3

[140] Moyer VA. Screening for primary hypertension in children and adolescents: U.S. Preventive Services Task Force recommendation statement. Pediatrics. 2013;159(9):613–9. 10.7326/0003-4819-159-9-201311050-00725. 10.7326/0003-4819-159-9-201311050-00725 24097285

[141] Krist AH, Davidson KW, Mangione CM, Barry MJ, Cabana M, Caughey AB, et al. Screening for high blood pressure in children and adolescents. JAMA. 2020;324(18):1878. 10.1001/jama.2020.20122. 33170248 10.1001/jama.2020.20122

[142] Urbina EM, de Ferranti S, Steinberger J. Observational studies may be more important than randomized clinical trials: Weaknesses in US Preventive Services Task Force recommendation on blood pressure screening in youth. Hypertension. 2014;63(4):638–40. 10.1161/HYPERTENSIONAHA.113.02662. 24366081 10.1161/HYPERTENSIONAHA.113.02662

[143] Li Y, Haseler E, McNally R, Sinha MD, Chowienczyk PJ. A meta-analysis of the haemodynamics of primary hypertension in children and adults. J Hypertens. 2023;41(2):212–9. 10.1097/hjh.0000000000003326. 36583348 10.1097/HJH.0000000000003326 PMC9799046

[144] Blumenthal S, Epps RP, Heavenrich R, Lauer RM, Lieberman E, Mirkin B, et al. Report of the Task Force on blood pressure control in children. Pediatrics. 1977;59(5):797–820. 859728

[145] New York State Education Department. Health examination guidelines for schools November 2022. https://www.p12.nysed.gov/sss/documents/school-health-examinations.pdf. Accessed 12 March 2024.

[146] Meng Y, Magnussen CG, Wu F, Juonala M, Buscot MJ, Pahkala K, et al. Impact of within-visit systolic blood pressure change patterns on blood pressure classification: The Cardiovascular Risk in Young Finns Study. Eur J Prev Cardiol. 2022;29(16):2090–8. 10.1093/eurjpc/zwac108. 35653303 10.1093/eurjpc/zwac108

[147] Urbina EM, Khoury PR, Bazzano L, Burns TL, Daniels S, Dwyer T, et al. Relation of blood pressure in childhood to self-reported hypertension in adulthood. Hypertension. 2019;73(6):1224–30. 10.1161/hypertensionaha.118.12334. 31067199 10.1161/HYPERTENSIONAHA.118.12334 PMC6510248

[148] Urbina EM, Daniels SR, Sinaiko AR. Blood pressure in children in the 21st century: What do we know and where do we go from here? Hypertension. 2023;80(8):1572–9. 10.1161/hypertensionaha.122.19455. 37278234 10.1161/HYPERTENSIONAHA.122.19455 PMC10524445

[149] Zhang Y, Yang L, Hou Y, Zhao M, Bovet P, Xi B. Performance of the simplified american academy of pediatrics table to screen elevated blood pressure in children. JAMA Pediatr. 2018;172(12):1196–8. 10.1001/jamapediatrics.2018.1923. 30105363 10.1001/jamapediatrics.2018.1923 PMC6583017

[150] Yang L, Hou Y, Zhao M, Bovet P, Xi B. Simplified blood pressure tables based on different height percentiles for screening elevated blood pressure in children. J Hypertens. 2019;37(2):292–6. 10.1097/hjh.0000000000001880. 30067249 10.1097/HJH.0000000000001880

[151] Dehmer SP, Maciosek MV, LaFrance AB, Flottemesch TJ. Health benefits and cost-effectiveness of asymptomatic screening for hypertension and high cholesterol and Aspirin counseling for primary prevention. Ann Fam Med. 2017;15(1):23–36. 10.1370/afm.2015. 28376458 10.1370/afm.2015 PMC5217841

[152] Wang YC, Cheung AM, Bibbins-Domingo K, Prosser LA, Cook NR, Goldman L, et al. Effectiveness and cost-effectiveness of blood pressure screening in adolescents in the United States. J Pediatr. 2011;158(2):257–64.e1–7. 10.1016/j.jpeds.2010.07.058. 20850759 10.1016/j.jpeds.2010.07.058 PMC4007283

[153] Padula WV, Kreif N, Vanness DJ, Adamson B, Rueda JD, Felizzi F, et al. Machine learning methods in health economics and outcomes research-the PALISADE checklist: A good practices report of an ISPOR Task Force. Value Health. 2022;25(7):1063–80. 10.1016/j.jval.2022.03.022. 35779937 10.1016/j.jval.2022.03.022

[154] Jafar TH, Gandhi M, de Silva HA, Jehan I, Naheed A, Finkelstein EA, et al. A community-based intervention for managing hypertension in rural South Asia. N Engl J Med. 2020;382(8):717–26. 10.1056/NEJMoa1911965. 32074419 10.1056/NEJMoa1911965

[155] Anand TN, Joseph LM, Geetha AV, Prabhakaran D, Jeemon P. Task sharing with non-physician health-care workers for management of blood pressure in low-income and middle-income countries: A systematic review and meta-analysis. Lancet Glob Health. 2019;7(6):e761–71. 10.1016/s2214-109x(19)30077-4. 31097278 10.1016/S2214-109X(19)30077-4 PMC6527522

[156] Tam HL, Wong EML, Cheung K. Educational program with text messaging for patients with hypertension in communities: A pilot randomized controlled trial. Asian Nurs Res (Korean Soc Nurs Sci). 2023. 10.1016/j.anr.2023.06.001. 37295501 10.1016/j.anr.2023.06.001

