# Supplementary material for: Pediatric Blood Pressure and Cardiovascular Health in Adulthood
Source: Curr Hypertens Rep. 2024 Jun 15;26(11):431–50. doi: 10.1007/s11906-024-01312-5 (PMC11455673; doi:10.1007/s11906-024-01312-5)
Supplement: Supplementary file 1 — Supplementary file1 (DOCX 168 KB) [file 11906_2024_1312_MOESM1_ESM.docx]

**Pediatric blood pressure and cardiovascular health in adulthood**

Yaxing Meng,^1,2^ Jonathan P. Mynard,^3,4,5^ Kylie J. Smith,^1,6^ Markus Juonala,^7,8^ Elaine M. Urbina,^9,10^ Teemu Niiranen,^11,12^ Stephen R. Daniels,^13^ Bo Xi,^14^ Costan G. Magnussen^1,2,15,16^

1. Baker Heart and Diabetes Institute, Melbourne, VIC, Australia.

2. Baker Department of Cardiometabolic Health, Faculty of Medicine, Dentistry and Health Sciences, University of Melbourne, Melbourne, VIC, Australia.

3. Heart Research Group, Murdoch Children's Research Institute, Parkville, VIC, Australia.

4. Department of Paediatrics, University of Melbourne, Parkville, VIC, Australia.

5. Department of Biomedical Engineering, University of Melbourne, Parkville, VIC, Australia.

6. Menzies Institute for Medical Research, University of Tasmania, Hobart, TAS, Australia.

7. Division of Medicine, Turku University Hospital, Turku, Finland.

8. Department of Medicine, University of Turku, Turku, Finland.

9. Heart Institute, Cincinnati Children's Hospital Medical Center, Cincinnati, OH, USA.

10. Department of Pediatrics, University of Cincinnati, Cincinnati, OH, USA.

11. Department of Public Health Solutions, Finnish Institute for Health and Welfare (THL), Helsinki, Finland.

12. Department of Internal Medicine, University of Turku and Turku University Hospital, Turku, Finland.

13. Department of Pediatrics, University of Colorado Anschutz Medical Campus, Aurora, CO, USA.

14. Department of Epidemiology, School of Public Health, Shandong University, Jinan, China.

15. Research Centre of Applied and Preventive Cardiovascular Medicine, University of Turku, Turku, Finland.

16. Centre for Population Health Research, University of Turku and Turku University Hospital, Turku, Finland.

**Address for correspondence:**

A/Prof. Costan G. Magnussen

Address: Baker Heart and Diabetes Institute, 75 Commercial Rd, Melbourne, VIC 3004, Australia.

E-mail: [Costan.Magnussen@baker.edu.au](mailto:Costan.Magnussen@baker.edu.au);

Phone: +61 (03) 8532 1111

**Supplement Text 1**

**Evidence-based lifestyle interventions**

The 2017 AAP guideline highlights the pivotal role of lifestyle modifications to prevent abnormal BP in youth[1]. This is supported by several short-term randomized trials, in which lifestyle interventions were effective in obtaining a lower BP level in youth. A meta-analysis of 10 randomized trials including 966 children and adolescents aged 8 to 16 years showed that interventions aimed at a lower salt intake (durations ranging from 2 weeks to 3 years) were associated with a 1.17 mmHg lower SBP and a 1.29 mmHg lower DBP[2]. Using data from another three randomized trials of 551 infants aged from 8 weeks to 6 months, meta-analysis showed that interventions aimed at a lower salt intake (median duration of 20 weeks) were associated with a 2.47 mmHg lower SBP in infancy (DBP: not reported)[2]. Data from the Dietary Intervention Study in Children showed that the dietary intervention group had a 2.3 mmHg lower SBP than the control group 9 years after termination of the 7-year intervention[3, 4]. Similarly, in the Special Turku Coronary Risk Factor Intervention Project, dietary counselling from infancy until 15 years of age was associated with, on average, a 1 mmHg lower SBP across all age-points[4].

The 2017 AAP guideline recommends promoting increased physical activity by engaging in 30-60 minutes of moderate to vigorous exercise at least three times per week[1], which is supported by a cluster-randomized controlled school-based trial (Action Schools! BC)[5]. Further, high-intensity interval-based training has emerged as a potentially more time-efficient approach to physical activity than conventional exercise programs in youth[6, 7]. Data from the Fitness für Kids project including 46 school-aged youth [aged mean (SD), 10.7 (0.6)] showed that the intervention group that underwent 3-months high-intensity interval training had a 6.13 mmHg lower SBP compared with the control group that underwent a traditional physical education curriculum[6]. Similar results were reported in a systematic review[7].

Obesity prevention programs in youth have also been shown to be favorably associated with BP levels[8-11]. A systematic review and meta-analysis of 23 randomized trials showed that losing weight in childhood was associated with, on average, a 1.64 mmHg lower SBP and 1.44 mmHg lower DBP[11]. Confirmatory findings were reported in a recent Swedish randomized trial, in which a 1-SD decrease in BMI was associated with 0.41 mmHg (95 % CI, 0.33, 0.49) lower SBP and 0.26 mmHg (95 % CI, 0.20, 0.32) lower DBP among obese youth[8].

Table S1. Risk of high blood pressure in adulthood among youth with high blood pressure.

| Publication | Sample | Age, years^a^ | | Follow-up, years | High-risk BP definition | | Finding(s) |
| --- | --- | --- | --- | --- | --- | --- | --- |
|  |  | Baseline | Follow-up |  | Childhood | Adulthood |  |
| **High-risk BP according to 2017 AAP in childhood, 2017 ACC/AHA in adulthood** | | | | | | | |
| *Bogalusa Heart Study* | | | | | | | |
| Du et al., 2019[12] | N=3940 (47 % male)  Race:  65 % white; 35 % black | 3–18 | 19–57 | 36 | SBP/DBP ≥95th age-, sex-, and height-specific percentile | SBP/DBP ≥130/80 mmHg, or with self-reported treatment of hypertension | RR (95 % CI)  1.66 (1.47-1.87) |
| **High-risk BP according to 2004 NHBPEP in childhood, 2017 ACC/AHA in adulthood** | | | | | | | |
| *The International Childhood Cardiovascular Cohort (i3C) Consortium* | | | | | | | |
| Urbina et al., 2019[13] | N=5035 (40 % male)  Race:  74% white, 17 % black, and 8 % other | 8–18 | 50 (5) | 47 | SBP≥90th percentile or SBP ≥120 mmHg | SBP ≥120 mmHg | OR (95 % CI)  For participants aged 8-11 years at baseline  2.0 (1.6, 2.5)  For participants aged 12-18 years at baseline  3.0 (2.2, 4.1) |
| *Bogalusa Heart Study* | | | | | | | |
| Du et al., 2019[12] | N=3940 (47 % male)  Race:  65 % white; 35 % black | 3–18 | 19–57 | 36 | SBP/DBP ≥95th age-, sex-, and height-specific percentile | SBP/DBP ≥130/80 mmHg, or with self-reported treatment of hypertension | RR (95 % CI)  1.71 (1.48, 1.98) |
| *Childhood Determinants of Adult Health Study* | | | | | | | |
| Kelly et al., 2015[14] | N=798 (47 % male) | Male:  12.0 (2.5)  Female:  11.9 (2.4) | Male:  32.0 (2.7)  Female:  31.8 (2.5) | 20 | SBP/DBP ≥90th age-, sex-, and height-specific percentile | SBP/DBP ≥120 /80mmHg or taking antihypertensive medication. | RR (95 % CI)  Male:  1.28 (1.13-1.46)  Female:  1.58 (1.08, 2.33) |
| **High-risk BP according to 2004 NHBPEP in childhood, 2018 ESC/ESH in adulthood** | | | | | | | |
| *Bogalusa Heart Study* | | | | | | | |
| Xi et al., 2017[15] | N=1225 (43 % male)  Race:  69 % white; 31 % black. | 10.9 (3.3) | 37.3 (4.5) | 27.1 | ≥95th age-, sex-, and height-specific percentile | SBP/DBP ≥140/90 mmHg or taking antihypertensive medicine | HR (95 % CI)  3.17 (1.99, 5.04) |
| *Cardiovascular Risk in Young Finns Study* | | | | | | | |
| Juhola et al., 2012[16] | N=2625 (46 % male) | 10.6 (5.0) | 24–45 | 21 or 27 | SBP/DBP ≥90th age-, sex-, and height-specific percentile | SBP/DBP ≥130/85 mmHg or taking antihypertensive medication | OR (95 % CI)  2.18 (1.82, 2.61) |
| Juhola et al., 2011[17] | N=2204 (49 % male) | 6–18 | 38–45 | 27 | SBP/DBP ≥90th percentiles by sex, age, and height | SBP/DBP ≥140/90 mm Hg or taking antihypertensive medicine | OR (95 % CI)  For participants aged 3, 6, 9 at baseline:  2.4 (1.5, 3.9);  For participants aged 12,15,18 at baseline:  2.3 (1.7, 3.0) |
| **High-risk BP according to simplified definitions^c^ in childhood, 2018 ESC/ESH in adulthood** | | | | | | | |
| *Bogalusa Heart Study* | | | | | | | |
| Xi et al., 2017[15] | N=1225 (43 % male)  Race:  69 % white; 31 % black. | 10.9 (3.3) | 37.3 (4.5) | 27 | Participants aged <12 years:  SBP/DBP ≥120/80 mmHg;  Participants aged ≥12 years:  SBP/DBP ≥130/85 mmHg. | SBP/DBP ≥140/90 mmHg or taking antihypertensive medicine | HR (95 % CI)  3.11 (1.83, 5.26) |
| *Amsterdam Growth and Health Study* | | | | | | | |
| Twisk et al.,1997[18] ^b^ | N=181 (46 % male) | 13.1 (0.8) | 27.1 (0.8) | 14 | SBP/DBP ≥126/82 mmHg  (upper 25th percentile) | SBP/DBP ≥140/90 mmHg | OR (95 % CI)  SBP: 4.0 (2.5, 6.5);  DBP: 4.8 (2.5, 9.4) |
| **High-risk BP according to non- standardized definition in childhood, 2018 ESC/ESH in adulthood** | | | | | | | |
| *Fels Longitudinal Study* | | | | | | | |
| Sun et al. 2007[19] | N=493 (49 % male) | 5–18 | Males with Mets:  45.2 (10.3)  Males without Mets:  34.6 (5.9)  Females with Mets:  47.3 (11.1)  Females without Mets:  34.0 (4.8) | Not reported | The age- and sex- specific least-squares means (absolute values not reported) | SBP/DBP ≥130/85 mmHg | Age at initial measurement, years,  OR (95 % CI):  Males:  5–7, 3.8 (1.5, 9.7)  8–13, 3.5 (1.5, 8.3)  14–18, 1.1 (0.5, 2.4)  Females:  5–7, 4.5 (1.1, 17.7)  8–13, 2.7 (1.0, 7.1)  14–18, 3.8 (1.2, 12.7) |
| **High-risk BP according to non- standardized definitions in childhood and adulthood** | | | | | | | |
| *Muscatine Study* | | | | | | | |
| Lauer et al. 1993[20] | N=2445 (48 % male) | 7–18 | 20–30 | Not reported | SBP >90th percentile among the observed participants;  DBP >90th percentile among the observed participants | SBP >90th percentile among the observed participants;  DBP >90th percentile among the observed participants | RR (95 % CI):  SBP: 2.4 (Not reported), P<0.001  DBP: 1.7 (Not reported), P<0.001 |
| *Odense Schoolchild Study* | | | | | | | |
| Lambrechtsen et al. 1999[21] | N=1369 (50 % male) | 8–10 | 19–21 | 11 | Not reported (appears to be upper quartile) | Not reported (appears to be upper quartile) | Not reported [OR for staying in the upper or lower quartile through the follow-up period ranged from 2.1 to 3.1 for SBP, and from 1.6 to 2.4 for DBP (95 % CI: Not reported)]. |
| *Bogalusa Heart Study* | | | | | | | |
| Shear et al. 1986[22] | N=1501 (48 % male)  Race: 2/3 white, 1/3 black | 2–14 | 10–22 | 8 | age-, sex-, and race-specific upper quartile | age-, sex-, and race-specific upper quartile | OR (95 % CI: Not reported)  SBP 2.5; DBP 2.0 |
|  |  | 5–17 | 10–22 | 5 |  |  | OR (95 % CI: Not reported)  SBP 4.3; DBP 3.2 |
|  |  | 7–19 | 10–22 | 3 |  |  | OR (95 % CI: Not reported)  SBP 5.8; DBP 3.6 |

^a^ Age represented as mean (standard deviation), or age range (minimum to maximum age).

^b^ This study used all available BP data to assess tracking coefficients, whereas other studies restricted to two time -points in childhood and adulthood.

^c^ Threshold values for high normal BP and hypertension are absolute values instead of sex-, age- and height-specific blood pressure percentiles.

Abbreviations: AAP, American Academy of Pediatrics; ACC/AHA, American College of Cardiology/American Heart Association; CI, confidence interval; DBP, diastolic blood pressure; ESC/ESH, European Society of Cardiology/European Society of Hypertension; HR, hazards ratio; Mets, metabolic syndrome; NHBPEP, National High Blood Pressure Education Program; OR, odds ratio; RR, relative risk; SBP, systolic blood pressure

Table S2. Overview of studies that have examined the cross-sectional association of youth blood pressure and target-organ damage.

| Publications | Sample | Age, years  Mean (SD)  or range | Findings |
| --- | --- | --- | --- |
| **Large artery stiffness** | | | |
| Haley et al., 2022[23] | N=382  60% males  63% White  16% Hispanic | 15.6 (1.8) | Participants were stratified by SBP into low (SBP <75th percentile, n=155), mid- (SBP ≥75th and <90th percentile, n=88), and high-risk categories (SBP ≥90th percentile, n=139), based on age-, sex-, and height-specific pediatric BP cut points.  PWV was higher in high-risk group than in the mid-risk group and the low-risk group [mean (SD) PWV: low-risk group, 4.83(0.69); mid-risk group 5.08 (0.76); high-risk group, 5.35 (0.92); P<0.05] |
| Lurbe et al., 2012[24] | N= 501  53% males | 12.6 (2.2) | For per 1-SD increase in SBP, PWV increased 0.329 m/s (95% confidence interval not reported). |
| Urbina et al.,2011[25] | N=723  34% males 40% Caucasian, | 10–23 | From normal BP to prehypertension to hypertension, a graded increase in arterial stiffness was observed: increased PWV [mean (SD), 5.75 (0.92), 6.38 (1.06), 7.12 (1.25) m⁄s, P<0.05], and increased augmentation index [mean (SD), 0.69 (11.52), 3.89 (10.21), 9.35 (10.62) %, P<0.05] |
| **IMT** | | | |
| Urbina et al.,2011[25] | N=723  34% males 40% Caucasian | 10–23 | Participants with hypertension had higher carotid IMT than their counterparts with normal BP [mean (SD), 0.53 (0.10) VS. 0.50 (0.09), mm, P<0.05] |
| Urbina et al.,2009[26] | N=446  39% males  35% Caucasian | 10–24 | SBP was positivity associated with carotid IMT with adjustment for age, sex, race, lipids, and body mass index (β for 1 mmHg increase, 1.31 mm, 95% confidence interval not reported, P<0.05) |
| **LV parameters** | | | |
| Urbina et al., 2019[27] | N=303  55% male  63% white | 11–19 | The prevalence of LV hypertrophy (LV mass index≥38.6 g/m^2.7^) increased with increasing clinical categories of BP (normotensive 13%, elevated BP 21%, hypertension 27%, P≤0.03).  Higher SBP associated with increased odds of LV hypertrophy, but a similar association was not observed for DBP [per 1-mmHg higher BP, odds ratio (95% confidence interval) for SBP was 1.8 (1.27, 2.62), DBP not reported).  The 90th percentile for SBP resulted in the best balance between sensitivity and specificity for predicting LV hypertrophy: When individual SBP percentiles were examined, the 90th percentile of SBP had a reasonably high specificity (0.75) with minimal loss of sensitivity (0.44) compared with lower SBP percentiles. |
| Falkner et al., 2013[28] | N=301  52% males | 13–18 | Compared with those with average SBP <75th percentile, adolescents with SBP from the 75th to 90th percentile had higher LV mass index (33.2 vs 38.7 gm/m^2.7^, P<0.001) and greater prevalence of LV hypertrophy (18% vs 43%, P <0.001), independent of obesity.  Elevated BP (defined as SBP/DBP>120/80 mmHg) was associated with increased odds of LV hypertrophy [Odds ratio (95% confidence interval), 2.92 (1.57, 5.43)]. |
| Urbina et al.,2011[25] | N=723  34% males  40% Caucasian | 10–23 | From normal BP to prehypertension to hypertension, there was a graded increase in LV mass index [mean (SD), 32.8 (8.9), 35.9 (9.8), 40.7 (11.2), g ⁄m^2.7^, P<0.05] |
| **Kidney damage** | | | |
| Haley et al., 2022[23] | N=382  60% males  63% White  16% Hispanic | 15.6 (1.8) | Participants were stratified by SBP into low (SBP <75th percentile, n=155), mid- (SBP ≥80th and <90th percentile, n=88), and high-risk categories (SBP ≥90th percentile, n=139), based on age-, sex-, and height-specific pediatric BP cut points. There was no statistically significant differences in urine albumin to creatinine ratio across these SBP groups. |
| Matjuda et al., 2020[29] | N=303  45% males | 6–9 | SBP was positively but weakly correlated with urinary albumin to creatinine ratio level (correlation coefficient: r = 0.206; p < 0.05). |
| Falkner et al., 2013[28] | N=301  52% males | 13–18 | No statistically significant difference in urinary microalbumin excretion between high versus normal BP groups. |
| **Cognitive performance** | | | |
| Lamballais et al., 2018[30] | N=5853  47% males | 6.1 (0.5) | DBP associated with standardized nonverbal intelligence [For per 1-SD, β (95% confidence interval), -0.030 (-0.054; -0.005)] after adjusting for child and maternal covariates. This association remained after excluding the top DBP decile [-0.042 (-0.075; -0.009)], suggesting that the relation holds in normotensives.  Such associations were not observed for SBP. |
| Ditto et al., 2006[31] | N=88 boys | 14 (0) | Boys with SBP above the median had poorer verbal learning scores. |
| Lande et al., 2003[32] | N=5077  52% males | 6–16 | Elevated SBP (SBP ≥90th percentile) was associated with lower digit span scores (β, -0.612, standard error, 0.277, P value, 0.032) than those with normal SBP (SBP <90th percentile), with adjustment for race, sex, parental education, poverty status, medication use, antihistamine use, general health status, lead level, body mass index, and heart rate. |

Abbreviations: BP, blood pressure; DBP, diastolic blood pressure; IMT, carotid intima media thickness; LV, left ventricular; PWV, pulse wave velocity; SBP, systolic blood pressure; SD, standard deviation.

Table S3. Overview of studies that have examined the longitudinal association of youth blood pressure and target-organ damage.

| Publications | | Sample | | Age, years  Mean (SD) or range | | | | Findings | |
| --- | --- | --- | --- | --- | --- | --- | --- | --- | --- |
|  |  |  |  | Baseline | | Follow-up | |  |  |
| **Large artery stiffness** | | | | | | | | | |
| Fan et al., 2022[33] ^a^ | | N=900  (41 % male)  35 % black;  65 % white | | 4–19 | | 49 (5) | | Cumulative SBP [calculated as the area under the curve of several BP measurements (the number of visits ranged from 5-16)] from childhood to adulthood was associated with adulthood aortic-femoral PWV [for per 1-SD increase, β (95 % CI): white, 0.387 (0.313, 0.460) m/s, black, 0.455 (0.355, 0.555) m/s, male, 0.501 (0.407, 0.595) m/s, female, 0.369 (0.294, 0.444) m/s] adjusted for cumulative BMI, adult heart rate, smoking, drinking, and antihypertensive and lipid-lowering medications. | |
| Aatola et al., 2017[34] ^b^ | | N=1540  (45 % male) | | 6–18 | | 30–45 | | Compared to those with normal BP in childhood and adulthood, increased risk of high adult PWV (defined as values≥ age-, sex-, and heart rate-specific 80th percentile) was observed among participants with persistently elevated BP in both childhood (defined as ≥ age-sex-and height-specific 90th percentile by the NIH/NHLBI) and adulthood (defined as SBP/DBP ≥120/80 mmHg) [RR (95 % CI), 3.18 (2.22, 4.55)] and participants with normal childhood BP but elevated adulthood BP [2.64 (1.79, 3.88)]. In contrast, individuals with elevated BP in childhood but not in adulthood did not have statistically significantly increased risk of high PWV [1.26 (0.80, 1.99)]. | |
| Yan et al., 2017[35] ^c^ | | N=1252 (55.3 % male) | | 11.6 (3.7) | | 34.5 (3.7) | | Childhood SBP and the change in SBP from childhood to adulthood (calculated as adulthood SBP values minus childhood SBP values), were both associated with adulthood high PWV (defined as ≥ age- and sex-specific 75th percentile), including carotid-femoral PWV and brachial-ankle PWV. Results were consistent among both sexes.  For both sexes, cumulative SBP [calculated as the area under the curve of several BP measurements (the number of visits ranged from 2-9, the mean number of visits was 4.5)] from childhood to adulthood over mean (SD) 22.9 (0.6) years was associated with high carotid-femoral PWV [for per 1-SD increase, OR (95 % CI): males, 2.20 (1.77, 2.74), females, 2.02 (1.54, 2.64)] and high brachial-ankle PWV [males, 2.49 (2.01, 3.07), females, 3.10 (2.32, 4.16)] in adulthood with adjustment for length to follow-up, adulthood LDL-C, HDL-C, TG, alcohol consumption, physical activity and family history of CVD. | |
| Chu et al., 2017[36] ^d^ | | N=259  (56 % male) | | 6–15 | | 32–41 | | Childhood SBP associated with brachial-ankle PWV in adulthood in a multivariable model that included sex, family history of hypertension, adulthood SBP, glucose, and serum uric acid. | |
| Ceponiene et al., 2015[37] ^e^ | | N=380  (44 % male) | | 12–13 | | 48–49 | | For both sexes, adolescent SBP was not associated with adulthood carotid-femoral PWV with adjustment for adulthood SBP, BMI, HDL-C, LDL-C, smoking, and educational levels [for per 1-SD increase, β (95 % CI), males: 0.045 (-0.372, 0.462) m/s; females: -0.165 (-0.409, 0.079) m/s]. | |
| Li et al., 2014[38]^a^ | | N=680  (43 % male)  27 % black; 73 % white. | | 4–17 | | 24–43 | | Of the observed risk factors in childhood (SBP, BMI, HDL-C, LDL-C, TG, smoking), SBP (per Z-score increase) was the only childhood risk factor significantly associated with adult brachial-ankle PWV [β (SE), 34.27 (9.28) cm/s].  In sex and race stratified analyses, childhood SBP (per Z-score increase) was only associated with adulthood brachial-ankle PWV among white females [β (SE), white males, 27.36 (13.97) cm/s; white females, 38.18 (13.89) cm/s; black males, 22.58 (29.20) cm/s; black females, 51.71 (30.67) cm/s] (the interaction term of SBP and sex/race was statistically nonsignificant). | |
| Liang et al. 2014[39]^c^ | | N=1259 (55 % male) | | 6–18 | | 30–42 | | Compared to those with normal BP, children with elevated BP (defined as SBP/DBP ≥age- and sex-specific 80th percentile) had increased odds of high carotid-femoral PWV (defined as ≥ age- and sex-specific 80th percentile) [OR (95 % CI), 1.8 (1.3, 2.4)] with adjustment for age, sex, birth weight, and factors in adults (smoking, alcohol consumption, physical inactivity, heart rate, obesity, hypertension, diabetes, dyslipidemia). | |
| Aatola et al., 2013[40] ^b^ | | N=1241 (45 % male) | | 6–15 | | 30–45 | | Elevated childhood BP associated with high adulthood arterial PWV (defined as values ≥age-, sex-, and heart rate-specific 80th percentile). The estimates were similar when the definition of elevated BP was age specific [RR (95 % CI), 1.5 (1.1, 2.0)], age- and sex-specific [1.6 (1.2, 2.2)], and age-, sex-, and height-specific [1.7 (1.2, 2.2)]. | |
| Ferreira et al., 2012[41] ^f^ | | N = 373  (% male not  reported) | | 13 | | 36 | | Individuals in the highest tertile of cD at age 36 years were characterized by higher SBP, DBP and MAP in adolescence (aged 13 years) and experienced larger increases in these measures between childhood and adulthood compared with those with the least stiff arteries at age 36 years. | |
| Aatola et al., 2010[42] ^b^ | | N=1691 (45 % male) | | 3–18 | | 30–45 | | In a multivariable model, childhood SBP load (assessed as the average of Z scores at the first three surveys) was associated with adulthood arterial PWV with adjustment for sex, childhood age, and glucose [for per 1-SD increase, β (SE), 0.08 (0.03) m/s]. | |
| Juonala et al., 2005[43] ^b^ | | N=2255  (45 % male) | | 3–18 | | 24–39 | | Childhood SBP was associated with cD [for per 1-SD increase, β (SE), -0.106 (0.017) %/10 mmHg] adjusted for childhood age, sex, and skinfold thickness.  The association of childhood SBP and adult cD was attenuated about a half (95 % CI not reported) and was not longer statistically significant (P=0.08) when adulthood SBP was also included in the model. | |
| Li et al., 2004[44]^a^ | | N=835  (44 % male).  28 % black  72 % white, | | 4–17 | | 24–44 | | Of the observed risk factors in childhood (SBP, BMI, HDL-C, LDL-C, TG, smoking), SBP was the only predictor for brachial-ankle PWV in adults (for per race-, sex-, and age-specific z scores increase: β (95 % CI),0.085 (not reported) m/s, P=0.021).  Cumulative SBP [calculated as the area under the curve of several BP measurements (76% participants had at least 6 measurements)] from childhood to adulthood over the mean 26.5 years was associated with brachial-ankle PWV in adults (for per race-, sex-, and age-specific Z scores increase: β (95 % CI), 0.299 (not reported) m/s, p<0.001). | |
| Oren et al., 2003[45]^g^ | | N=524  (46 % male) | | Males: 13.5 (1.1)  Females:  13.4 (1.1) | | Males: 28.2 (0.9)  Females:  28.2 (0.9) | | Childhood BP was not associated with carotid-femoral PWV [for per 10 mmHg increase: β (95 % CI), SBP, 0.03 (-0.09, 0.14) m/s; DBP, 0.01 (-0.13, 0.15) m/s; MAP, 0.02 (-0.13, 0.17)]. | |
| **Intima-media thickness** | | | | | | | | | |
| Fan et al., 2022[33] ^a^ | | N=900  (41 % male)  35% black;  65% white | | 4–19 | | 49 (5) | | Cumulative SBP [calculated as the area under the curve of several BP measurements (the number of visits ranged from 5-16)] from childhood to adulthood was associated with adulthood carotid IMT [for per 1-SD increase, β (95 % CI): white, 0.217 (0.117, 0.318) mm, black, 0.181 (0.060, 0.301) mm, male, 0.194 (0.066, 0.321) mm, female, 0.219 (0.123, 0.314) mm] adjusted for cumulative BMI, adult heart rate, smoking, drinking, and antihypertensive and lipid-lowering medications. | |
| Koskinen et al., 2019[46]^h^ | | N=5925  (54 % male) | | 3–18 | | 19–51 | | Childhood SBP provided the best predictive ability to adult carotid IMT, compared to other BP components (DBP or MAP) in childhood (area under the receiver operating characteristic curves, SBP, 0.677 vs DBP V Korotkoff phase, 0.669, P=0.006; or vs DBP IV Korotkoff phase, 0.670, P=0.004; or vs MAP V Korotkoff phase, 0.672, P=0.003;or vs MAP IV Korotkoff phase, 0.674, P=0.01). | |
| Koskinen et al., 2018[47]^h^ | | N=2893 (46 % male) | | 12–18 | | Not reported (mean follow-up, 23.4 years) | | Compared to those with normal BP in adolescence, participants with elevated BP (age-, sex-, and height-specific values according to the NIH/NHLBI guideline) had higher risk of high carotid IMT (≥ specific 90th percentile) [RR (95 % CI), high normal: 1.4 (1.0, 1.9), hypertension: 1.9 (1.3, 2.9)] in a multivariable model including sex, adolescence lipids and BMI. | |
| Hao et al., 2017[48]^i^ | | N=683  (50 % male).  52 % European American;  48 % African American | | 5–16 | | Not reported  (the duration of follow-up was 23 years) | | Trajectory groups in SBP from childhood to young adulthood [low-increasing groups: reference group; moderate-increasing: β (95 %), 0.019 (0.005, 0.032) mm; high-increasing: 0.051 (0.027, 0.074) mm] associated with carotid IMT in young adulthood in a multivariable model that included age, race, sex, BMI, father’s education level, SBP, and DBP. | |
| Yan et al., 2017[35]^c^ | | N=1252 (55.3 % male) | | 11.6 (3.7) | | 34.5 (3.7) | | Childhood SBP and the change in SBP from childhood to adulthood (calculated as adulthood SBP values minus childhood SBP values), were both associated with adulthood high carotid IMT (defined as ≥ age- and sex- specific 75th percentile). Results were consistent among both sexes.  For both sexes, cumulative SBP [calculated as the area under the curve of several BP measurements (the number of visits ranged from 2-9, the mean number of visits was 4.5)] from childhood to adulthood over mean (SD) 22.9 (0.6) years was associated with high carotid IMT [for per 1-SD increase, OR (95 % CI): males, 1.25 (1.04, 1.50), females, 1.53 (1.18, 1.98)] in adulthood with adjustment for length to follow-up, adulthood LDL-C, HDL-C,TG, alcohol consumption, physical activity, and family history of CVD. | |
| Oikonen et al., 2016[49]^b^ | | N=1927  (46 % male) | | 3–24 | | 30–45 | | Compared with a single measurement, multiple measurements of elevated BP in childhood did not improve prediction of adult elevated carotid IMT (>90th percentile or carotid plaques) (area under the receiver operating characteristic curves, single elevated BP, 0.59 vs. two elevated BP in childhood, 0.59, P = 0.82). | |
| Ceponiene et al., 2015[37]^e^ | | N=380  (44 % male) | | 12–13 | | 48–49 | | Adolescent SBP was associated with adulthood carotid IMT in females but not in males [for per 1-SD increase, β (95 % CI), males: 0.002 (-0.020, 0.023) mm; females: 0.013 (0.002, 0.025) mm] with adjustment for adulthood SBP, BMI, HDL-C, LDL-C, smoking, and educational levels. | |
| Liang et al. 2014[39]^c^ | | N=1259 (55 % male) | | 6–18 | | 30–42 | | Compared to those with normal BP, children with elevated BP (defined as SBP/DBP ≥age- and sex-specific 80th percentile) had increased odds of high carotid IMT (defined as ≥ age- and sex-specific 80th percentile) [OR (95 % CI), 1.4 (1.0, 1.9)] with adjustment for age, sex, birth weight, and factors in adults (smoking, alcohol consumption, physical inactivity, heart rate, obesity, hypertension, diabetes, and dyslipidemia). | |
| Juhola et al., 2013[50]^h^ | | N=4210 (45 % male) | | 4–18 | | 23–46 | | Compared to those with normal BP in childhood and adulthood, an increased risk of high adult carotid IMT (defined as ≥ age-, sex-, race-, and cohort-specific 90th percentile) was observed in participants with persistently elevated BP in both childhood (defined as ≥ age-sex-and height-specific 90th percentiles by the NIH/NHLBI) and adulthood (defined as SBP/DBP≥ 120/80 mmHg) [RR (95 % CI), 1.82 (1.47, 2.38)] and participants with normal childhood BP but elevated adulthood BP [1.57 (1.22, 2.02)]. In contrast, individuals with elevated BP in childhood but not in adulthood did not have statistically significantly increased risk of high adult carotid IMT [1.24 (0.92, 1.67)]. | |
| Juonala et al., 2010[51]^h^ | | N=4380 (46 % male) | | 3–18 | | 20–45 | | Childhood SBP obtained at 6 [for per 1-SD increase, β (SE), 0.102 (0.033) mm], 9 [0.041 (0.025) mm], 12 [0.078 (0.023) mm],15 [0.063 (0.022) mm] and 18 [0.064 (0.027) mm] years of age was associated with carotid IMT in adulthood in a multivariable model that included lipids and BMI.  DBP levels were not associated with carotid IMT in any of the age groups (data not shown). | |
| Li et al., 2007[52]^a^ | | N=868  (42 % male)  29 % black;  71 % white | | 4–17 | | 25–44 | | Childhood SBP was associated with adulthood carotid IMT in white females (for per 1-SD increase, β=0.132 (95 % CI not reported); P=0.025) and in black males (β=0.241; P=0.031), but not in white males (β=0.082; P=0.202) and black females (β=0.115; P=0.171) with adjustment for childhood BMI and HDL-C, LDL-C and TG. | |
| Raitakari et al., 2003[53]^b^ | | N=2229  (45 % male) | | 3–18 | | 24–39 | | SBP measured in adolescence (aged 12-18 years) was significantly associated with adult carotid IMT with adjustment for age, sex, and childhood LDL-C, BMI, and smoking status [β (SE), 0.013 (0.003) mm]. This association remained significant when adjusted for adulthood SBP. | |
| Davis et al., 2001[54]^j^ | | N=725  (48 % male) | | 8–18 | | 33–42 | | Childhood BP was correlated with adult carotid IMT among females (Spearman correlation, SBP,0.15, DBP, 0.10) but not among males (SBP, 0.10, DBP, 0.06) in univariable models. Among females, BP was not correlated with carotid IMT in a multivariable model that included childhood cholesterol levels and BMI. | |
| **Carotid plaque** | | | | | | | | | |
| Koskinen et al., 2020[55]^b^ | | N=2653 (44 % male) | | 6–18 | | 24–45 | | Elevated cumulative SBP (calculated as the area under the curve) in childhood was not associated with the presence of carotid plaque in adulthood in a multivariate model that included adult SBP, child and adult dyslipidemia, overweight, smoking, and family history of coronary heart disease [RR (95 % CI), 1.43 (0.92, 2.22)]. | |
| **Coronary artery calcification** | | | | | | | | | |
| Allen et al. 2014[56]^k^ | | N= 4681 | | 18–30 | | Not reported  (the duration of follow-up was 25 years) | | Compared with the low-stable SBP trajectory group, trajectories with elevated SBP levels had increased odds of having a coronary artery calcification score of 100 HU or greater [OR (95 % CI), moderate-stable, 1.44 (0.83, 2.49); moderate-increasing, 1.86 (0.91,3.82); elevated-stable, 2.28 (1.24, 4.18); elevated-increasing, 3.70 (1.66, 8.20)]. Results were similar for DBP trajectories. [OR (95 % CI): low-stable, reference group; moderate-stable, 1.57 (0.91, 2.71); moderate-increasing, 1.97 (0.77, 5.03); elevated-stable, 2.09 (1.13, 3.89); elevated-increasing, 2.20 (0.81, 6.01)]. | |
| Hartiala et al., 2012[57]^b^ | | N=589  (45 % male) | | 12–18 | | 30–46 | | Adolescent SBP levels associated with coronary artery calcification in adulthood independently of changes in SBP from childhood to adulthood [for per 1-SD increase, OR (95 % CI), 1.38 (1.08, 1.77)]. | |
| Mahoney et al., 1996[58]^j^ | | N=384  (51 % male) | | 8–18 | | 29–37 | | Elevated childhood BP (defined as upper decile) was not associated with the presence of coronary artery calcification in adulthood (OR, SBP: males, 1.3; females, 0.5; DBP: males, 1.0; females, 1.2. All P values >0.05. 95 % CI, not reported). | |
|  | |  | |  | |  | | **Endothelial function** | |
| Juonala et al., 2006[59]^b^ | | N=2109 (44 % male) | | 3–18 | | 24–39 | | Childhood (3–9 years) SBP was not associated with adult FMD among males or females.  Adolescent (12–18 years) SBP was negatively associated with adult FMD among males but not females. For adolescent males, a significant association remained with adjustment for adolescent risk factors (BMI, lipids, smoking, insulin, birth weight) and brachial diameter [per 1 mmHg increase, β (SE), -0.049 (0.016) %].  Childhood and adolescent DBP were not associated with adult FMD. | |
|  | |  | |  | |  | | **LV parameters** | |
| Heiskanen et al., 2021[60]^b^ | | N=1864 (45 % male) | | 6–18 | | 34–49 | | Cumulative SBP in childhood (the area under the curve derived from SBP measurements between the ages 6 and 18) was not associated with LV mass. | |
| Liu et al., 2020[61]^a^ | | N=1108 (41.9 % male)  35 % black;  65 % white | | 10 (3) | | 48.2 (5.3) | | SBP and DBP of childhood, and cumulative SBP and DBP (total area under the curve) from childhood to adulthood were associated with LVMI, adjusted for BMI, race, sex, and age. | |
| Hao et al., 2017[48]^i^ | | N=683  (50 % male).  52 % European American;  48 % African American | | 5–16 | | Not reported | | Trajectory groups in SBP from childhood to young adulthood [low-increasing groups: reference group; moderate-increasing: β (95 % CI), 2.785 (0.448, 5.121) g/m^2^; high-increasing: 7.451 (3.644, 11.257) g/m^2^] associated with LVMI in young adulthood in a multivariable model that included age, race, sex, BMI, father’s education level, SBP, and DBP. | |
| Liang et al. 2014[39]^c^ | N=1259 (55 % male) | | 6–18 | | 30–42 | | Compared to those with normal BP, children with elevated BP (defined as SBP/DBP ≥age- and sex-specific 80th percentile) had increased odds of high LVMI (defined as ≥ age- and sex-specific 80th percentile) [OR (95 % CI), 1.4 (1.0, 1.9)] with adjustment for age, sex, birth weight, and factors in adulthood (smoking, alcohol consumption, physical inactivity, heart rate, obesity, hypertension, diabetes, dyslipidemia). | |  |
| Lai et al., 2014[62]^a^ | N=1061 (43 % male)  32 % black;  68 % white | | 10 (3) | | 24–46 | | SBP in childhood, as well as total area under the curve from childhood to adulthood (cumulative BP) and incremental area under the curve from childhood to adulthood were all significantly associated with LVMI [for per 1-SD increase, β (95 % CI), childhood SBP, 0.08 (0.01, 0.14) g/m^2.7^; total area under the curve, 0.14 (0.09, 0.20) g/m^2.7^; incremental area under the curve, 0.10 (0.05, 0.16) g/m^2.7^] and LV hypertrophy [for per 1-SD increase, OR (95 % CI), childhood SBP, 1.27 (1.04, 1.54); total area under the curve, 1.47 (1.20,1.80); incremental area under the curve, 1.43 (1.19, 1.72)], adjusted for BMI, race, sex, and age.  Results were similar for DBP (data not shown). | |  |
| Magnussen et al., 2014[63]^l^ | N=181  (53 % male) | | 9, 12, 15 | | 31.3 (2.6) | | The correlation between adult LVMI and childhood DBP (r=0.65, P=0.02 versus r=0.16, P=0.07; P for difference=0.05) was stronger among those with a family history of coronary heart disease than in those without. Results for SBP were not reported. | |  |
| Toprak  et al., 2008[64]^a^ | N=824  (41 % male).  31 % black  69 % white, | | 5–18 | | 24–44 | | Childhood DBP was a significant predictor of adult concentric LV hypertrophy vs. normal geometry independent of other childhood risk factors including adiposity [for per 1-mmHg increase, OR (95 % CI), 1.14 (1.03, 1.26)]. | |  |
| Li et al., 2004[65]^a^ | N=467  (39 % male)  29 % black  71 % white | | 4–17 | | 20–38 | | Cumulative SBP from childhood to adulthood (total area under the curve) was associated with LVMI, adjusted for BMI, lipids, race, sex, and age. | |  |
| **Kidney damage** | | | | | | | | |  |
| Leiba et al., 2017[66]^m^ | N=2.19 million Israeli military recruits | | 16–19 | | Not reported (Median follow-up: 16.8 years) | | Prehypertensive youth BP (90th to 95th percentiles or 120–139/80–89 mmHg) had increased incidence of end-stage renal disease with a hazard ratio of 1.32 (95% CI, 1.11, 1.58) adjusted for year of birth, age at examination, sex, BMI, education, socioeconomic status, and country of origin.  Hypertension in youth (blood pressure above the 95th percentile or above 140/90 mmHg) was associated with a hazard ratio of 1.44 (95% CI, 1.17, 1.79).  A spline model demonstrated a nadir of risk at SBP values as low as 94 mmHg. | |  |
| **Cognitive Performance** | | | | | | | | |  |
| Rovio et al., 2017[67]^b^ | N=2026 | | 6–24 | | 34–49 | | SBP in childhood (6-12 years), adolescence (12-18 years), or young adulthood (18-24 years) was associated with poorer performance in PAL-test (indicating visual and episodic memory and visuospatial associative learning) in mid-adulthood, adjusting for age and sex [for per 1-SD, β (standard error), -0.058 (0.023), -0.067 (0.026), -0.097 (0.030), respectively].  Cumulative burden of SBP from childhood to young adulthood (calculated as area under the curve) was associated with poorer performance in PAL-test in mid-adulthood, adjusting for age, sex, total cholesterol, smoking, childhood family income, adulthood antihypertension and dyslipidemia medications, and diagnoses of cardiovascular diseases and diabetes mellitus [for per 1-SD, β (standard error), –0.064 (0.028)]. | |  |

^a^ The Bogalusa Heart Study

^b^ The Cardiovascular Risk in Young Finns Study

^c^ The Beijing Blood Pressure Cohort

^d^ The Hanzhong Adolescent Hypertension Cohort

^e^ The Kaunas Cardiovascular Risk Cohort Study

^f^ The Amsterdam Growth and Health Longitudinal Study

^g^ The Atherosclerosis Risk in Young Adults Study

^h^ The International Childhood Cardiovascular Cohort Consortium

^i^ The Georgia Stress and Heart Study

^j^ The Muscatine Heart Study

^k^ The Coronary Artery Risk Development in Young Adults Study

^l^ The Childhood Determinants of Adult Health Study

^m^ Not reported

Abbreviations: BMI, body mass index; cD, carotid distensibility; CI, confidence interval; IMT, intima-media thickness; CVD, cardiovascular disease; FMD, flow-mediated dilatation; HDL-C, high density lipoprotein cholesterol; LDL-C, low density lipoprotein cholesterol; LV, left ventricular; LVMI, left ventricular mass index; MAP, mean arterial pressure; NIH/NHLBI, National Institutes of Health’s National Heart, Lung, and Blood Institute; OR, odds ratio; PWV, pulse wave velocity; RR, relative risk; SBP, systolic blood pressure; SD, standard deviation; SE, standard error; TG, triglycerides.

Table S4. Treatment goals and indication of pharmacological intervention

|  | 2017 American Academy of Paediatrics guideline | 2016 European Society of Hypertension guideline |
| --- | --- | --- |
| **Treatment goals** | | |
| Hypertension without comorbidities | Age <16 years:  <95th percentile  Age ≥16 years:  <140/90 mmHg | Age <13 years:  <90th percentile or <130/80 mmHg, whichever is lower  Age ≥13 years:  <130/80 mmHg |
| Hypertension with diabetes | Age <16 years:  <90th percentile  Age ≥16 years:  <130/80 mmHg | Age <13 years:  <90th percentile or <130/80 mmHg, whichever is lower  Age ≥13 years:  <130/80 mmHg |
| Hypertension with chronic kidney disease |  |  |
| Without proteinuria | Age <16 years:  <75th percentile  Age ≥16 years:  <130/80 mmHg | All youth:  <50th percentile mean arterial pressure by ambulatory blood pressure monitoring |
| With proteinuria | Age <16 years:  <50th percentile  Age ≥16 years:  <125/75 mmHg |  |
| **Indication of pharmacological intervention** | | |
| Failed nonpharmacological therapy | Children who remain hypertension  despite a trial of lifestyle modifications | Children who remain hypertension despite nonpharmacological therapy for about 1 year. |
| Symptomatic hypertension | Yes | Yes |
| Stage 2 hypertension | Yes | Yes |
| Hypertension with diabetes or chronic kidney disease | Yes | Yes |
| Hypertension mediated target organ damage (e.g., left ventricular hypertrophy) | Yes | Yes |
| Secondary hypertension | Yes | Yes |

**References**

1. Flynn JT, Kaelber DC, Baker-Smith CM, Blowey D, Carroll AE, Daniels SR et al. Clinical practice guideline for screening and management of high blood pressure in children and adolescents. Pediatrics. 2017;140(3):e20171904. doi:10.1542/peds.2017-1904.

2. He FJ, MacGregor GA. Importance of salt in determining blood pressure in children: Meta-analysis of controlled trials. Hypertension. 2006;48(5):861-9. doi:10.1161/01.HYP.0000245672.27270.4a.

3. Dorgan JF, Liu L, Barton BA, Deshmukh S, Snetselaar LG, Van Horn L et al. Adolescent diet and metabolic syndrome in young women: Results of the Dietary Intervention Study in Children (DISC) follow-up study. J Clin Endocrinol Metab. 2011;96(12):e1999-2008. doi:10.1210/jc.2010-2726.

4. Niinikoski H, Jula A, Viikari J, Rönnemaa T, Heino P, Lagström H et al. Blood pressure is lower in children and adolescents with a low-saturated-fat diet since infancy: The Special Turku Coronary Risk Factor Intervention Project. Hypertension. 2009;53(6):918-24. doi:10.1161/hypertensionaha.109.130146.

5. Reed KE, Warburton DE, Macdonald HM, Naylor PJ, McKay HA. Action Schools! BC: A school-based physical activity intervention designed to decrease cardiovascular disease risk factors in children. Prev Med. 2008;46(6):525-31. doi:10.1016/j.ypmed.2008.02.020.

6. Ketelhut S, Kircher E, Ketelhut SR, Wehlan E, Ketelhut K. Effectiveness of multi-activity, high-intensity interval training in school-aged children. Int J Sports Med. 2020;41(4):227-32. doi:10.1055/a-1068-9331.

7. Eddolls WTB, McNarry MA, Stratton G, Winn CON, Mackintosh KA. High-intensity interval training interventions in children and adolescents: A systematic review. Sports Med. 2017;47(11):2363-74. doi:10.1007/s40279-017-0753-8.

8. Hagman E, Danielsson P, Elimam A, Marcus C. The effect of weight loss and weight gain on blood pressure in children and adolescents with obesity. Int J Obes (Lond). 2019;43(10):1988-94. doi:10.1038/s41366-019-0384-2.

9. Seo YG, Lim H, Kim Y, Ju YS, Lee HJ, Jang HB et al. The effect of a multidisciplinary lifestyle intervention on obesity status, body composition, physical fitness, and cardiometabolic risk markers in children and adolescents with obesity. Nutrients. 2019;11(1). doi:10.3390/nu11010137.

10. Son WM, Sung KD, Bharath LP, Choi KJ, Park SY. Combined exercise training reduces blood pressure, arterial stiffness, and insulin resistance in obese prehypertensive adolescent girls. Clin Exp Hypertens. 2017;39(6):546-52. doi:10.1080/10641963.2017.1288742.

11. Cai L, Wu Y, Wilson RF, Segal JB, Kim MT, Wang Y. Effect of childhood obesity prevention programs on blood pressure: A systematic review and meta-analysis. Circulation. 2014;129(18):1832-9. doi:10.1161/circulationaha.113.005666.

12. Du T, Fernandez C, Barshop R, Chen W, Urbina EM, Bazzano LA. 2017 pediatric hypertension guidelines improve prediction of adult cardiovascular outcomes. Hypertension. 2019;73(6):1217-23. doi:10.1161/hypertensionaha.118.12469.

13. Urbina EM, Khoury PR, Bazzano L, Burns TL, Daniels S, Dwyer T et al. Relation of blood pressure in childhood to self-reported hypertension in adulthood. Hypertension. 2019;73(6):1224-30. doi:10.1161/hypertensionaha.118.12334.

14. Kelly RK, Thomson R, Smith KJ, Dwyer T, Venn A, Magnussen CG. Factors affecting tracking of blood pressure from childhood to adulthood: The Childhood Determinants of Adult Health Study. J Pediatr. 2015;167(6):1422-8. doi:10.1016/j.jpeds.2015.07.055.

15. Xi B, Zhang T, Li S, Harville E, Bazzano L, He J et al. Can pediatric hypertension criteria be simplified? Hypertension. 2017;69(4):691-6. doi:10.1161/hypertensionaha.116.08782.

16. Juhola J, Oikonen M, Magnussen CG, Mikkilä V, Siitonen N, Jokinen E et al. Childhood physical, environmental, and genetic predictors of adult hypertension. Circulation. 2012;126(4):402-9. doi:10.1161/circulationaha.111.085977.

17. Juhola J, Magnussen CG, Viikari JS, Kähönen M, Hutri-Kähönen N, Jula A et al. Tracking of serum lipid levels, blood pressure, and body mass index from childhood to adulthood: The Cardiovascular Risk in Young Finns Study. J Pediatr. 2011;159(4):584-90. doi:10.1016/j.jpeds.2011.03.021.

18. Twisk JW, Kemper HC, van Mechelen W, Post GB. Tracking of risk factors for coronary heart disease over a 14-year period: A comparison between lifestyle and biologic risk factors with data from the Amsterdam Growth and Health Study. Am J Epidemiol. 1997;145(10):888-98. doi:10.1093/oxfordjournals.aje.a009048.

19. Sun SS, Grave GD, Siervogel RM, Pickoff AA, Arslanian SS, Daniels SR. Systolic blood pressure in childhood predicts hypertension and metabolic syndrome later in life. Pediatrics. 2007;119(2):237-46. doi:10.1542/peds.2006-2543.

20. Lauer RM, Clarke WR, Mahoney LT, Witt J. Childhood predictors for high adult blood pressure. The Muscatine Study. Pediatr Clin North Am. 1993;40(1):23-40. doi:10.1016/s0031-3955(16)38478-4.

21. Lambrechtsen J, Rasmussen F, Hansen H, Jacobsen I. Tracking and factors predicting rising in ‘tracking quartile’ in blood pressure from childhood to adulthood: Odense Schoolchild Study. J Hum Hypertens. 1999;13(6):385-91. doi:10.1038/sj.jhh.1000836.

22. Shear CL, Burke GL, Freedman DS, Berenson GS. Value of childhood blood pressure measurements and family history in predicting future blood pressure status: Results from 8 years of follow-up in the Bogalusa Heart Study. Pediatrics. 1986;77(6):862-9.

23. Haley JE, Woodly SA, Daniels SR, Falkner B, Ferguson MA, Flynn JT et al. Association of blood pressure-related increase in vascular stiffness on other measures of target organ damage in youth. Hypertension. 2022;79(9):2042-50. doi:10.1161/hypertensionaha.121.18765.

24. Lurbe E, Torro I, Garcia-Vicent C, Alvarez J, Fernández-Fornoso JA, Redon J. Blood pressure and obesity exert independent influences on pulse wave velocity in youth. Hypertension. 2012;60(2):550-5. doi:10.1161/hypertensionaha.112.194746.

25. Urbina EM, Khoury PR, McCoy C, Daniels SR, Kimball TR, Dolan LM. Cardiac and vascular consequences of pre-hypertension in youth. J Clin Hypertens (Greenwich). 2011;13(5):332-42. doi:10.1111/j.1751-7176.2011.00471.x.

26. Urbina EM, Kimball TR, McCoy CE, Khoury PR, Daniels SR, Dolan LM. Youth with obesity and obesity-related type 2 diabetes mellitus demonstrate abnormalities in carotid structure and function. Circulation. 2009;119(22):2913-9. doi:10.1161/circulationaha.108.830380.

27. Urbina EM, Mendizábal B, Becker RC, Daniels SR, Falkner BE, Hamdani G et al. Association of blood pressure level with left ventricular mass in adolescents. Hypertension. 2019;74(3):590-6. doi:10.1161/hypertensionaha.119.13027.

28. Falkner B, DeLoach S, Keith SW, Gidding SS. High risk blood pressure and obesity increase the risk for left ventricular hypertrophy in African-American adolescents. J Pediatr. 2013;162(1):94-100. doi:10.1016/j.jpeds.2012.06.009.

29. Matjuda EN, Sewani-Rusike CR, Anye SNC, Engwa GA, Nkeh-Chungag BN. Relationship between high blood pressure and microalbuminuria in children aged 6-9 years in a South African population. Children (Basel). 2020;7(9). doi:10.3390/children7090131.

30. Lamballais S, Sajjad A, Leening MJG, Gaillard R, Franco OH, Mattace-Raso FUS et al. Association of blood pressure and arterial stiffness with cognition in 2 population-based child and adult cohorts. J Am Heart Assoc. 2018;7(21):e009847. doi:10.1161/jaha.118.009847.

31. Ditto B, Séguin JR, Tremblay RE. Neuropsychological characteristics of adolescent boys differing in risk for high blood pressure. Ann Behav Med. 2006;31(3):231-7. doi:10.1207/s15324796abm3103_4.

32. Lande MB, Kaczorowski JM, Auinger P, Schwartz GJ, Weitzman M. Elevated blood pressure and decreased cognitive function among school-age children and adolescents in the United States. J Pediatr. 2003;143(6):720-4. doi:10.1067/s0022-3476(03)00412-8.

33. Fan B, Zhang T, Li S, Yan Y, Fan L, Bazzano L et al. Differential roles of life-course cumulative burden of cardiovascular risk factors in arterial stiffness and thickness. Can J Cardiol. 2022;38(8):1253-62. doi:10.1016/j.cjca.2022.03.009.

34. Aatola H, Koivistoinen T, Tuominen H, Juonala M, Lehtimäki T, Viikari JSA et al. Influence of child and adult elevated blood pressure on adult arterial stiffness: The Cardiovascular Risk in Young Finns Study. Hypertension. 2017;70(3):531-6. doi:10.1161/hypertensionaha.117.09444.

35. Yan Y, Hou D, Liu J, Zhao X, Cheng H, Xi B et al. Childhood body mass index and blood pressure in prediction of subclinical vascular damage in adulthood: Beijing Blood Pressure Cohort. J Hypertens. 2017;35(1):47-54. doi:10.1097/hjh.0000000000001118.

36. Chu C, Dai Y, Mu J, Yang R, Wang M, Yang J et al. Associations of risk factors in childhood with arterial stiffness 26 years later: The Hanzhong Adolescent Hypertension Cohort. J Hypertens. 2017;35 Suppl 1:S10-s5. doi:10.1097/hjh.0000000000001242.

37. Ceponiene I, Klumbiene J, Tamuleviciute-Prasciene E, Motiejunaite J, Sakyte E, Ceponis J et al. Associations between risk factors in childhood (12-13 years) and adulthood (48-49 years) and subclinical atherosclerosis: The Kaunas Cardiovascular Risk Cohort Study. BMC cardiovascular disorders. 2015;15:89. doi:10.1186/s12872-015-0087-0.

38. Li S, Chen W, Yun M, Fernandez C, Krousel-Wood M, Webber L et al. Sex and race (black-white) differences in the relationship of childhood risk factors to adulthood arterial stiffness: The Bogalusa Heart Study. Am J Med Sci. 2014;348(2):101-7. doi:10.1097/maj.0000000000000264.

39. Liang Y, Hou D, Shan X, Zhao X, Hu Y, Jiang B et al. Cardiovascular remodeling relates to elevated childhood blood pressure: Beijing Blood Pressure Cohort Study. Int J Cardiol. 2014;177(3):836-9. doi:10.1016/j.ijcard.2014.11.013.

40. Aatola H, Magnussen CG, Koivistoinen T, Hutri-Kähönen N, Juonala M, Viikari JS et al. Simplified definitions of elevated pediatric blood pressure and high adult arterial stiffness. Pediatrics. 2013;132(1):e70-6. doi:10.1542/peds.2012-3426.

41. Ferreira I, Van De Laar RJ, Prins MH, Twisk JW, Stehouwer CD. Carotid stiffness in young adults: A life course analysis of its early determinants. The Amsterdam Growth and Health Longitudinal Study. Hypertension. 2012;59(1):54-61. doi:10.1161/hypertensionaha.110.156109.

42. Aatola H, Hutri-Kähönen N, Juonala M, Viikari JSA, Hulkkonen J, Laitinen T et al. Lifetime risk factors and arterial pulse wave velocity in adulthood: The Cardiovascular Risk in Young Finns Study. Hypertension. 2010;55(3):806-11. doi:10.1161/hypertensionaha.109.145102.

43. Juonala M, Järvisalo MJ, Mäki-Torkko N, Kähönen M, Viikari JSA, Raitakari OT. Risk factors identified in childhood and decreased carotid artery elasticity in adulthood: The Cardiovascular Risk in Young Finns Study. Circulation. 2005;112(10):1486-93. doi:10.1161/circulationaha.104.502161.

44. Li S, Chen W, Srinivasan SR, Berenson GS. Childhood blood pressure as a predictor of arterial stiffness in young adults: The Bogalusa Heart Study. Hypertension. 2004;43(3):541-6. doi:10.1161/01.HYP.0000115922.98155.23.

45. Oren A, Vos LE, Uiterwaal CS, Gorissen WH, Grobbee DE, Bots ML. Adolescent blood pressure does not predict aortic stiffness in healthy young adults. The Atherosclerosis Risk in Young Adults (ARYA) Study. J Hypertens. 2003;21(2):321-6. doi:10.1097/00004872-200302000-00023.

46. Koskinen J, Juonala M, Dwyer T, Venn A, Petkeviciene J, Čeponienė I et al. Utility of different blood pressure measurement components in childhood to predict adult carotid intima-media thickness. Hypertension. 2019;73(2):335-41. doi:10.1161/hypertensionaha.118.12225.

47. Koskinen J, Juonala M, Dwyer T, Venn A, Thomson R, Bazzano L et al. Impact of lipid measurements in youth in addition to conventional clinic-based risk factors on predicting preclinical atherosclerosis in adulthood: International Childhood Cardiovascular Cohort Consortium. Circulation. 2018;137(12):1246-55. doi:10.1161/circulationaha.117.029726.

48. Hao G, Wang X, Treiber FA, Harshfield G, Kapuku G, Su S. Blood pressure trajectories from childhood to young adulthood associated with cardiovascular risk: Results from the 23-year longitudinal Georgia Stress and Heart Study. Hypertension. 2017;69(3):435-42. doi:10.1161/hypertensionaha.116.08312.

49. Oikonen M, Nuotio J, Magnussen CG, Viikari JS, Taittonen L, Laitinen T et al. Repeated blood pressure measurements in childhood in prediction of hypertension in adulthood. Hypertension. 2016;67(1):41-7. doi:10.1161/HYPERTENSIONAHA.115.06395.

50. Juhola J, Magnussen CG, Berenson GS, Venn A, Burns TL, Sabin MA et al. Combined effects of child and adult elevated blood pressure on subclinical atherosclerosis: The International Childhood Cardiovascular Cohort Consortium. Circulation. 2013;128(3):217-24. doi:10.1161/circulationaha.113.001614.

51. Juonala M, Magnussen CG, Venn A, Dwyer T, Burns TL, Davis PH et al. Influence of age on associations between childhood risk factors and carotid intima-media thickness in adulthood. Circulation. 2010;122(24):2514-20. doi:10.1161/circulationaha.110.966465.

52. Li S, Chen W, Srinivasan SR, Tang R, Bond MG, Berenson GS. Race (black-white) and gender divergences in the relationship of childhood cardiovascular risk factors to carotid artery intima-media thickness in adulthood: The Bogalusa Heart Study. Atherosclerosis. 2007;194(2):421-5. doi:10.1016/j.atherosclerosis.2006.08.026.

53. Raitakari OT, Juonala M, Kähönen M, Taittonen L, Laitinen T, Mäki-Torkko N et al. Cardiovascular risk factors in childhood and carotid artery intima-media thickness in adulthood. JAMA. 2003;290(17):2277-83. doi:10.1001/jama.290.17.2277.

54. Davis PH, Dawson JD, Riley WA, Lauer RM. Carotid intimal-medial thickness is related to cardiovascular risk factors measured from childhood through middle age. The Muscatine Study. Circulation. 2001;104(23):2815-9. doi:10.1161/hc4601.099486.

55. Koskinen JS, Kytö V, Juonala M, Viikari JSA, Nevalainen J, Kähönen M et al. Childhood risk factors and carotid atherosclerotic plaque in adulthood: The Cardiovascular Risk in Young Finns Study. Atherosclerosis. 2020;293:18-25. doi:10.1016/j.atherosclerosis.2019.11.029.

56. Allen NB, Siddique J, Wilkins JT, Shay C, Lewis CE, Goff DC et al. Blood pressure trajectories in early adulthood and subclinical atherosclerosis in middle age. JAMA. 2014;311(5):490-7. doi:10.1001/jama.2013.285122.

57. Hartiala O, Magnussen CG, Kajander S, Knuuti J, Ukkonen H, Saraste A et al. Adolescence risk factors are predictive of coronary artery calcification at middle age: The Cardiovascular Risk in Young Finns Study. Journal of the American College of Cardiology. 2012;60(15):1364-70. doi:10.1016/j.jacc.2012.05.045.

58. Mahoney LT, Burns TL, Stanford W, Thompson BH, Witt JD, Rost CA et al. Coronary risk factors measured in childhood and young adult life are associated with coronary artery calcification in young adults: The Muscatine Study. J Am Coll Cardiol. 1996;27(2):277-84. doi:10.1016/0735-1097(95)00461-0.

59. Juonala M, Viikari JS, Rönnemaa T, Helenius H, Taittonen L, Raitakari OT. Elevated blood pressure in adolescent boys predicts endothelial dysfunction: The Cardiovascular Risk in Young Finns Study. Hypertension. 2006;48(3):424-30. doi:10.1161/01.Hyp.0000237666.78217.47.

60. Heiskanen JS, Hernesniemi JA, Ruohonen S, Hutri-Kähönen N, Kähönen M, Jokinen E et al. Influence of early-life body mass index and systolic blood pressure on left ventricle in adulthood – The Cardiovascular Risk in Young Finns Study. Ann Med. 2021;53(1):160-8. doi:10.1080/07853890.2020.1849785.

61. Liu Y, Yan Y, Jiang T, Li S, Guo Y, Fernandez C et al. Impact of long‐term burden of body mass index and blood pressure from childhood on adult left ventricular structure and function. J Am Heart Assoc. 2020;9(16):e016405. doi:10.1161/jaha.120.016405.

62. Lai CC, Sun D, Cen R, Wang J, Li S, Fernandez-Alonso C et al. Impact of long-term burden of excessive adiposity and elevated blood pressure from childhood on adulthood left ventricular remodeling patterns: The Bogalusa Heart Study. J Am Coll Cardiol. 2014;64(15):1580-7. doi:10.1016/j.jacc.2014.05.072.

63. Magnussen CG, Dwyer T, Venn A. Family history of premature coronary heart disease, child cardio-metabolic risk factors and left ventricular mass. Cardiol Young. 2014;24(5):938-40. doi:10.1017/s1047951113001571.

64. Toprak A, Wang H, Chen W, Paul T, Srinivasan S, Berenson G. Relation of childhood risk factors to left ventricular hypertrophy (eccentric or concentric) in relatively young adulthood (from the Bogalusa Heart Study). Am J Cardiol. 2008;101(11):1621-5. doi:10.1016/j.amjcard.2008.01.045.

65. Li X, Li S, Ulusoy E, Chen W, Srinivasan SR, Berenson GS. Childhood adiposity as a predictor of cardiac mass in adulthood. Circulation. 2004;110(22):3488-92. doi:10.1161/01.cir.0000149713.48317.27.

66. Leiba A, Twig G, Vivante A, Skorecki K, Golan E, Derazne E et al. Prehypertension among 2.19 million adolescents and future risk for end-stage renal disease. J Hypertens. 2017;35(6):1290-6. doi:10.1097/hjh.0000000000001295.

67. Rovio SP, Pahkala K, Nevalainen J, Juonala M, Salo P, Kähönen M et al. Cardiovascular risk factors from childhood and midlife cognitive performance: The Young Finns Study. J Am Coll Cardiol. 2017;69(18):2279-89. doi:10.1016/j.jacc.2017.02.060.
